# Exploring Pharmacists’ Perceptions of Their Current Role in Mental Health Trusts in England: A Qualitative Study

**DOI:** 10.3390/healthcare13202602

**Published:** 2025-10-16

**Authors:** Atta Abbas Naqvi, Muhammad Umair Khan, Hung Nguyen, Lee Karim, Asha Said, Adaora Nnadi

**Affiliations:** 1 School of Pharmacy, University of Reading, Whiteknights Campus, Reading RG6 6UR, UK; 2 Aston Pharmacy School, College of Health and Life Sciences, Aston University, Birmingham B4 7ET, UK; 3 Department of Kinesiology and Health Sciences, University of Waterloo, Kitchener, ON N2L 3G1, Canada; 4 Birmingham and Solihull Mental Health Trust, Birmingham B13 8QY, UK

**Keywords:** mental health, pharmacists, current opinion, pharmaceutical services, qualitative research, England

## Abstract

**Aim:** This study assessed how pharmacists perceive the impact of their role in the mental health (MH) services in two National Health Service (NHS) Trusts in England and their views on this service. **Methods:** An interview-based study was conducted from September to December 2023 on Microsoft Teams^®^ by interviewing the pharmacists involved in MH services in Berkshire Healthcare NHS Foundation Trust & the Birmingham and Solihull Mental Health Trust (BSMHFT) in England. Interviews were conducted using a semi-structured interview guide containing questions related to pharmacists’ roles, activities, perceptions about the service, and future recommendations. Transcripts were prepared and analysed using thematic analysis. The study was approved by the ethics committee of the School of Pharmacy at the University of Reading and was registered as a service evaluation with both Trusts. **Results:** A total of 11 participants attended the interviews. Most of the participants self-identified as women (*n* = 9), worked between 25 and 40 h on average weekly (*n* = 8), and had training in MH (*n* = 7). Few (*n* = 4) had work experience >20 years. Four themes emerged: (1) Roles and responsibilities—pharmacists play a vital role in medication management, clinical decision-making, and patient counselling; (2) satisfaction and positive impacts—a high job satisfaction derived from improved patient outcomes and effective multidisciplinary collaboration was reported; (3) challenges and barriers—stigma, role ambiguity, limited training in mental health, and institutional challenges (workload, funding, etc.), were identified; participants also expressed scepticism about the readiness of newly qualified prescriber pharmacists; (4) recommendations—participants advocated for enhanced MH content in pharmacy curricula, societal awareness and de-stigmatisation. **Conclusions:** Pharmacists viewed their role as integral to providing MH services; however, progress is impeded by challenges such as stigma, fragmented care, training gaps, and staffing shortages. It seemed unclear at the moment how the new prescriber-ready pharmacists will contribute to services. Additional findings from primary-care settings would provide a collective account of the current roles of pharmacists and their potential in MH.

## 1. Introduction

The World Health Organisation (WHO) describes mental health as a state that enables an individual to manage life’s stresses, recognise their skills and abilities, learn, and work to contribute to their community. Good mental health is not only desirable in its own right but also contributes to individual productivity [1]. Mental health (MH) conditions carry a huge global disease burden [2]. Figures from the WHO report that approximately 970 million people globally suffer from common MH conditions such as anxiety and depression. One in every six years lived with a disability is attributed to MH conditions, and people with severe MH issues live 10 to 20 years less than the general population in their region [1]. In terms of economic implications, productivity loss is enormous and goes beyond the direct cost of treatment. Common mental health conditions, such as depression and anxiety, are estimated to result in the loss of 12 billion productive days and cost about USD 1 trillion to the global economy yearly, owing to lost productivity [3]. Many individuals experience stigma and other social challenges that may exacerbate productivity losses due to MH.

Mental health conditions are one of the top causes of disabilities in the UK. It has been reported that 1 in 4 adults in the UK experiences a mental health condition, while 1 in 5 adults experience common MH conditions such as anxiety and depression [4,5]. In terms of gender identity, about 1 in 5 women and 1 in 7 men have common mental health conditions [6]. It is worthwhile mentioning that people from ethnically diverse communities are more likely to suffer from these conditions [5]. People from White ethnic backgrounds were more likely to receive treatment [6], while those from Black ethnic backgrounds were less likely to receive treatment [5,6]. It was also reported in the Adult Psychiatric Morbidity Survey 2014 that 1 in 3 people with common MH conditions had sought treatment for their condition [6]. The economic cost of mental health conditions in the UK was estimated at £105 billion in 2016 [7], rising to £300 billion in 2022 [8,9]. The NHS England spent £12.13 billion in 2024/5, which was slightly higher than the £11.31 billion spent during the 2023/4 yearly cycle [10].

Pharmacists are the healthcare professionals entrusted with the responsibility of providing person-centred care [2]. Their role in mental healthcare is recognised by both international and local pharmacy associations [11,12]. While the role of the pharmacist is frequently discussed, there is comparatively less emphasis on measuring its effectiveness and impact. Traditionally, pharmacists have been involved in managing prescriptions and collaborating with the members of the allied health teams in treatment [2]. New roles for pharmacists include leading specialist clinics, administering injectable medications, conducting consultations, independent prescribing, and contributing to academic and training roles [2,13,14,15,16]. A few studies have explored the roles of pharmacists in supporting patients with common MH conditions and assessed their impact. Several studies have shown the promising contributions of pharmacists in areas such as counselling [17], supplementary and independent prescribing [18,19], educational activities [20], medicines management [21], and consultations [22]. However, these studies focused on a single role or intervention rather than a broad focus and did not capture pharmacists’ views. Currently, there is no clear description of the range of activities pharmacists can perform in NHS mental health services [8]. In addition, it is important to understand their views about the service and its future, especially new regulations about prescriber-ready pharmacists at the point of graduation are being rolled out. This multicentre study was designed to explore the breadth of roles undertaken by pharmacists in secondary-care NHS mental health settings to identify their responsibilities and capture their views on the service and its future.

## 2. Methods

### 2.1. Aims and Objectives

This study aimed to assess the current roles of pharmacists in mental health services in the National Health Service (NHS) and how they perceive their impact. The objectives of this study were to evaluate the pharmacists’ perceptions of their roles, their impact, pharmacists’ satisfaction and confidence with related tasks, and their views on the future of the service.

### 2.2. Study Design and Venues

A qualitative interview-based study was conducted from 15 September to 31 December 2023. Interviews were conducted online on Microsoft Teams^®^ by involving pharmacists working in Prospect Park Hospital (PPH), a mental health facility affiliated with the Royal Berkshire NHS Foundation Trust in Reading, and Birmingham and Solihull Mental Health Trust (BSMHFT) in Birmingham, England.

### 2.3. Participants and Recruitment

The target segment included pharmacists. All pharmacists who are registered with the General Pharmaceutical Council (GPhC), currently working in mental health services in the two trusts, and have access to Microsoft Teams^®^, were included. Pharmacists who did not have access to Microsoft Teams^®^, and those who did not consent to participate or withdrew participation, were excluded. A census approach was used, and all pharmacists at the two Trusts were invited. The participants at PPH were recruited by an academic member at the Reading School of Pharmacy who was also employed with the hospital. Also, the participants at the BSMHFT were recruited by an investigator who also worked at the Trust. The interviews were conducted by another researcher who did not have any relationship with the participants.

### 2.4. Research Instrument

The study used a semi-structured interview guide that contained 9 questions, and each question had at least 1 prompt. The questions were related to the research objectives, i.e., pharmacist’s role, daily activities within mental health service, level of accomplishment, challenges and barriers, future recommendations, etc. The interview guide was pilot-tested before use. This was done by conducting a mock interview involving two investigators to check for feasibility, duration, appropriateness, and cultural sensitivity of the questions, etc. In addition, a demographic information form was also used to gather participants’ information. It contained questions on age, gender identity, work experience of pharmacists, qualifications, training in mental health, and years of work as a pharmacist in the UK. The demographic form and the interview guide are available as a Supplementary File S1.

### 2.5. Data Collection and Analysis

The interviews were conducted by an investigator who self-identified as female and studied Master of Pharmacy (MPharm) degree. The researcher was supported by an investigator who self-identified as male, had a PhD qualification, and had over 10 years of work experience in academia and in conducting qualitative research. The duration of the interview was about 30–40 min. The interviews were conducted on Microsoft Teams^®^ and were recorded to facilitate the preparation of transcripts. The transcripts were cleaned, anonymised, and sent to the participants for validation. The recording was deleted once a participant confirmed the contents of the transcript. The interviews were conducted during the working day, during participants’ lunch hours. Therefore, they were reimbursed for giving their time. No repeat interviews were conducted.

The interview transcripts were independently coded by two researchers, and a codebook was subsequently prepared. The document is available as a Supplementary File S2. It was validated by another researcher through peer examination [23]. Thematic analysis was conducted using an inductive approach and followed the Braun and Clarke’s framework [24] as described by Ahmed and colleagues [25]. This resulted in the generation of themes supported by codes backed with anonymised quotes. Similar themes were grouped to form a major theme. Microsoft Word^®^ was used to facilitate the analysis and writing process. A qualitative analysis document was prepared. The document is available as a Supplementary File S3. Semantic linkages were developed and illustrated using Microsoft Visio^®^. The data from the demographic forms were analysed using IBM SPSS version 25. Descriptive statistics, sample counts, and frequency were reported. The quantitative raw dataset for demographic variables is available as a Supplementary File S4. The reporting of this work follows the COREQ (COnsolidated criteria for REporting Qualitative research) guidelines [26].

### 2.6. Ethics and Informed Consent

The participants were informed that participation was voluntary and that they could withdraw from the interview at any time without any consequences. The study was approved by the Research Ethics Committee at the School of Pharmacy, University of Reading (SREC 35/2023). In addition, this study was registered as a service evaluation by the Royal Berkshire NHS Foundation Trust (ID number 12073) and the Birmingham and Solihull Mental Health NHS Foundation Trust (Service Evaluation number SE0400).

## 3. Results

A total of 11 participants attended the interviews. Most of the participants self-identified as women (*n* = 9, 81.8%). Almost a third of participants (*n* = 3, 27.3%) were either less than 30 years old or between 31 and 40 years old. More than a third (*n* = 4, 36.4%) had either an MPharm or an independent prescriber qualification. More than half (*n* = 6, 54.5%) worked at BSMHFT. Most participants (*n* = 8, 72.7%) worked between 25 and 40 h on average in a week in the healthcare setting. In addition to the primary setting, participants were asked if they worked in any other healthcare setting, and only 2 (18.2%) worked in another healthcare setting that included a community pharmacy and a mental health hospital. A third of the participants (*n* = 4, 36.4%) had work experience of more than 20 years. Many participants (*n* = 7, 63.6%) had completed formal training and/or continuing education programmes in mental health services and spent more than 60 h doing so (*n* = 5, 45.5%).

The notable pieces of training and continuing education programmes were certificates in mental health, Psych 1 and 2 Therapeutics from the College of Mental Health Pharmacy (CMHP), a diploma in mental health, an independent prescribing course, a clozapine 10 module certificate, Psych 1 and 2 courses, and a Postgraduate Certificate in Mental Health Therapeutics. The details are mentioned in Table 1.

### 3.1. Qualitative Findings

Qualitative findings revealed four major themes with sub-themes that had several codes. These codes were supported by participants’ quotes. These themes are explained as follows:

#### 3.1.1. Theme 1: Roles and Responsibilities

**Figure 1:**
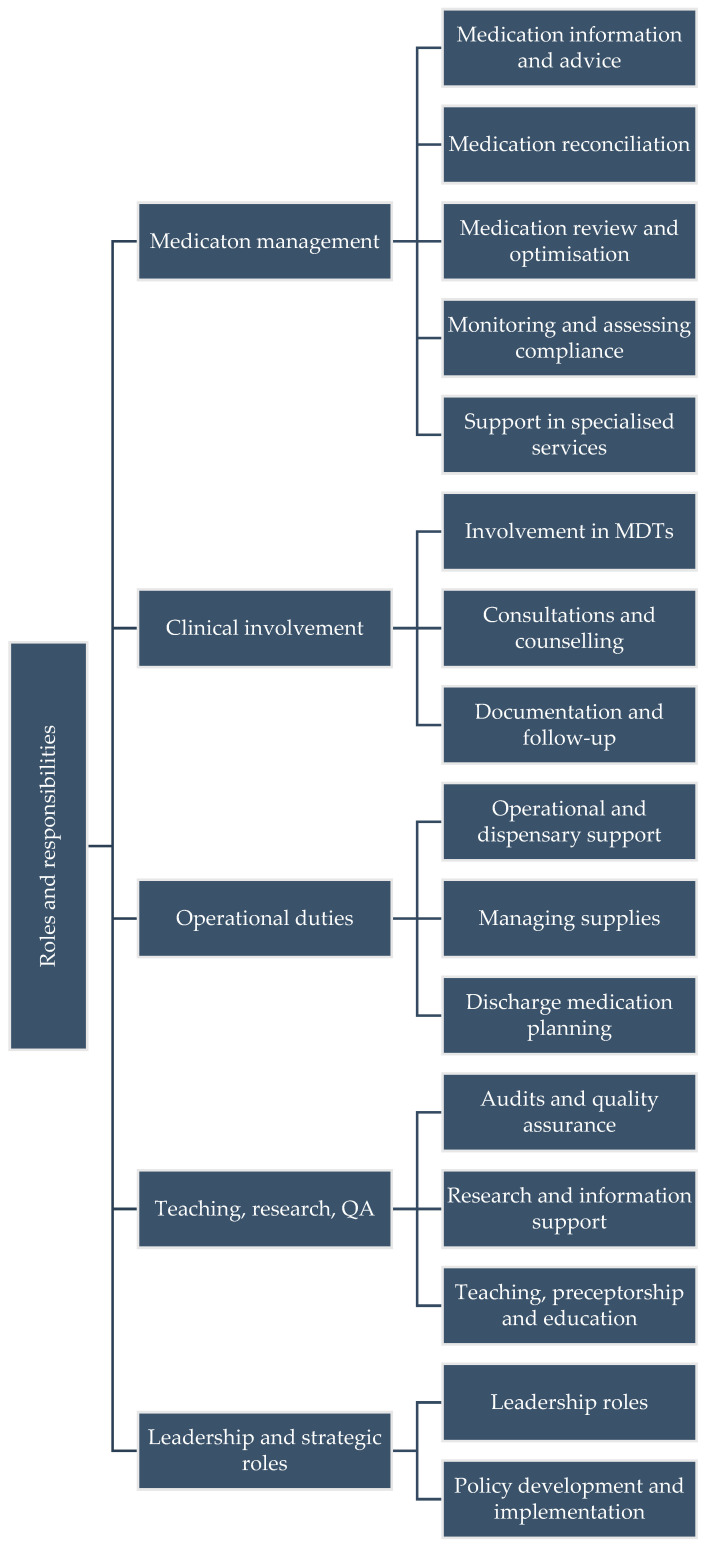
Theme diagram for Roles and Responsibilities of Pharmacists.

The first theme that emerged from the data was about the roles and responsibilities of pharmacists. This included core medication management, involvement in other clinical tasks, operational duties, teaching, research, quality assurance, and leadership and strategic roles (Figure 1).

##### Medication Management

Pharmacists play a key role in providing medication information and advice to both patients and healthcare professionals. They address medication-related inquiries, ranging from choices of use of medication (such as antidepressants) to complex medication decisions:

*I would answer queries from consultants in the public of our outpatient services regarding specific medication choices in patients with complex physical and mental health background.*

*(P2)*

Pharmacists also perform medication reconciliation, ensuring patient safety and effective care. They verify the accuracy of medication lists and review drug interactions and appropriateness. They also prioritise timely reconciliation, aiming to complete the process within 24 to 48 h of patient admission. Thanks to this process, medications are appropriately prescribed and reduce the risk of errors, particularly for patients with complex medication regimens that involve the monitoring of ECGs, blood tests, and vital signs.

The role of medication review and optimisation means an assessment of medication suitability. Pharmacists review patients’ medication charts to assess compliance. They also review psychotropic medications for safety and optimal dosing. They also monitor side effects and collaborate with other healthcare providers to make necessary adjustments. They may suggest switching and reducing medication to ensure patients receive the most appropriate, safe, and effective treatment regimens.

In addition, pharmacists assess medication compliance, particularly for high-risk medications such as sedatives and antipsychotics, and medications requiring specific tests such as lithium (requiring regular blood tests).

*I’m responsible for running what we call an insight report which is a basic report which highlights all the high levels above 600 micrograms litre per every week and then we have to report those back to the clinicians directly and also document everything in the patient notes”*

*(P7)*

*If patient requires switch from a medication to another medication either because patient wishes to change to something else or the side effect profile is not there is not something that patient can tolerate and then we help with the consultant to come with the alternative plan*

*(P8)*

Pharmacists take charge of monitoring and assessing patient compliance. In the words of two participants:

*We have to assess their compliance to the medications and sometimes it requires seeing each patient a few times throughout their stay.*

*(P3)*

*Monitoring for new prescriptions and making sure that new prescriptions are clinically checked, professionally checked and appropriately ordered.*

*(P9)*

In critical settings such as psychiatric intensive care units and care homes, pharmacists perform outpatient prescriptions in specialised services such as memory clinics, pain management, and child mental health services. In these services, they manage high-risk medications such as clozapine and lithium.:

*Every day we’re dealing with clozapine, which is, as you are probably aware of, a very high-risk drug if it’s not properly monitored.*

*(P7)*

*If somebody is on lithium, there are certain requirements and monitoring is required that we have to prompt our team to do the regular blood test.*

*(P8)*

##### Clinical Involvement

In multidisciplinary teams, pharmacists engage in ad hoc and regular meetings to provide team members (doctors, nurses, social workers) with insights into patient care. Their involvement extends to internal pharmacy meetings that address barriers to safety and quality improvement:

*We review the guidelines and give recommendations as a team… Discussing treatment plans for patients, monitoring progress, and making recommendations with references.*

*(P4)*

Consultations and counselling are key components of a pharmacist’s clinical role. They regularly consult with patients to discuss treatment options, side effects, and high-risk medications (clozapine, lithium). They also offer psychoeducation to help patients understand their illness and treatment plans. One-on-one patient counselling sessions focus on medication adherence, side effects, and next steps in the treatment process. Some pharmacists proactively engage with patients to check if they have any concerns or questions.

*Sometimes also consultations to speak to patients about any adverse reactions, treatment choice, and explanations of their new medications, especially for high-risk medications like clozapine and lithium.*

*(P2)*

Documentation and follow-up are integral to ensuring ongoing patient care and medication adherence. In this regard, pharmacists maintain detailed records for each patient, ensuring continuity in care and patient progress. Pharmacists are involved in verifying that medications are appropriately prescribed and taken as intended. They also contact community mental health teams to make sure plans are followed.

*We call and check that medications are ordered and patients are taking them continuously…We contact community mental health teams to make sure plans are followed.*

*(P4)*

##### Operational Duties

Pharmacists ensure efficient pharmacy operations, thanks to which medication dispensing is smooth and safe. They assist with preparing medication, processing prescriptions, and ensuring accuracy. They also cover dispensary duties. Their responsibilities extend to on-call duties and oversight of medication storage and supply. Additionally, pharmacists lead medication supply logistics, process nurse supply requests, and ensure medication availability across wards. Additionally, pharmacists prepare medication for patients at their hospital discharge and are effectively involved in coordinating discharge plans (medication needs, counselling in advance) with the community mental health team to ensure continuity of care.

*I’ll be involved in the dispensary and making sure the dispensary is running properly and potentially accuracy checking dispensed medication.*

*(P9)*

*When I arrive at work, I checked to see if we got any discharges… if it’s a short-term leave, how many days? How many of this tablet do we need?*

*(P1)*

##### Involvement in Teaching, Research, and Quality Assurance

Pharmacists conduct regular audits of controlled drugs to ensure compliance with safety and quality standards, contributing to the continuous improvement of pharmacy practices and patient safety. Moreover, they research medication-related queries from medical staff and provide evidence-based support, using established guidelines:

*We’ll look at like evidence-based situations… NICE guidelines… recommendations from the RPS… Maybe we should try this.*

*(P1)*

This research-based approach allows pharmacists to provide recommendations *“with pros and cons to support decisions.” (P4)* Pharmacists also contribute to clinical trials in their institutions:

*I work as a teacher practitioner pharmacist and my role is split 50/50 between teaching at (mentions the university) and working in a mental health setting.*

*(P10)*

Especially, pharmacists provide educational support (mentorship, role-modelling, instructions) to students and trainees, contributing significantly to their professional development. Their monthly clinical supervision meeting serves as ongoing educational support for colleagues and other healthcare teams.

##### Leadership and Strategic Roles

Pharmacists play crucial leadership and strategic roles, contributing significantly to team management, coordination, and policy development within healthcare settings. Many pharmacists occupy leadership positions, overseeing teams of other healthcare professionals to ensure effective service delivery, workflows, and optimal function. Pharmacists also introduce new guidelines, medication practices, and new protocols for compliance.

*I’m a lead pharmacist, so I’m leading a team of other Band 7 pharmacists and technicians.*

*(P8)*

*And then I’m involved in a variety of the governance, and operational roles…pharmacological Therapies Committee, approval of policies and new drugs…governance processes within the Secure Care service.*

*(P9)*

#### 3.1.2. Theme 2: Positive Aspects and Satisfaction

**Figure 2:**
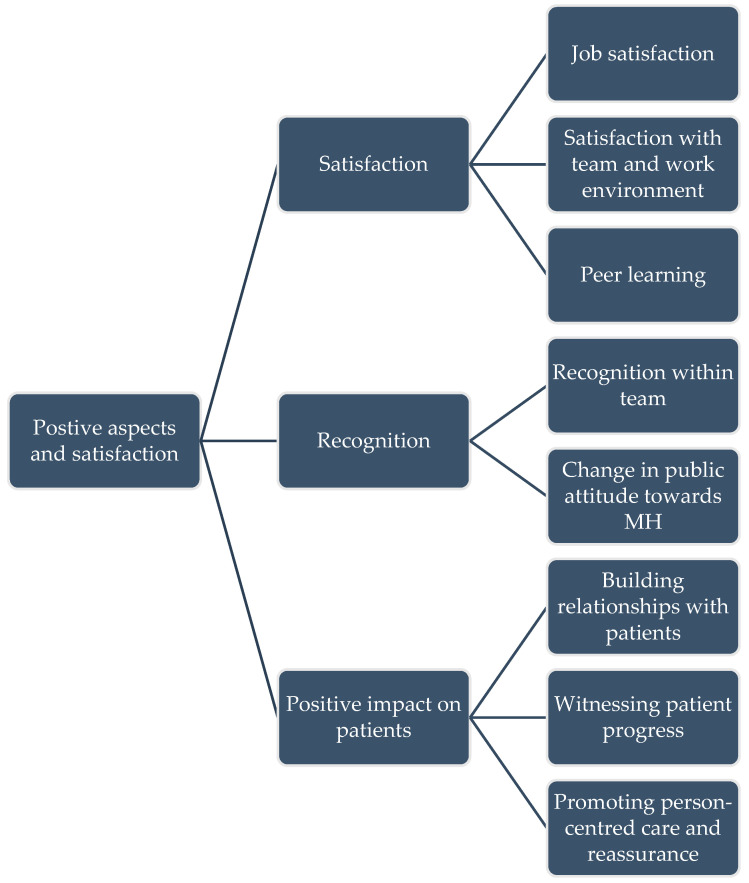
Theme diagram for Positive Aspects and Satisfaction.

The second theme that emerged was about the satisfaction of pharmacists with their roles and the positive aspects of the service. This included their personal satisfaction, their feeling of recognition, and the positive impact they bring on patients (Figure 2).

##### Satisfaction

Pharmacists’ satisfaction results from not only seeing patients’ improvement in mental health. Interviewed pharmacists expressed a sense of accomplishment because their presence on the team results in effective patient intervention:

*I get a lot more job satisfaction from working here… it’s really good to be able to sit down… and figure out something that will actually benefit them.*

*(P1)*

*When we have pharmacists on the team, the patient intervention is there much quicker and more efficiently.*

*(P8)*

Team collaboration may result in satisfaction because it fosters a sense of community and strong rapport. The sense of camaraderie extends beyond mere professional interactions. Additionally, peer learning forms a critical aspect of pharmacists’ professional development. Building rapport with colleagues may foster learning, practice insights, and navigation through complex situations. Regular supervision and open dialogue with senior colleagues ensure updates in clinical knowledge and skills.

##### Recognition of Mental Health and Pharmacy Services

Pharmacists working in mental health settings receive appreciation for their contributions to teams, of which they are proud. They envision an opportunity for professional growth within a team:

*I feel proud of my work because I feel like with mental health, not a lot of people, as pharmacists, want to do it.*

*(P10)*

Participants observed a positive shift in public attitudes towards mental health, particularly among younger generations:

*The attitudes in a younger generation are a bit changing.*

*(P2)*

*It’s better than before… 5–10 years ago to now is better. Most people do understand that mental health is like physical health.*

*(P4)*

Furthermore, the impact of the COVID-19 pandemic on mental health awareness was highlighted, with one participant stating:

*Since COVID, I think the whole British population are quite willing to talk about… the troubles that they’re going through.*

*(P6)*

##### Positive Impacts on Patients

Building personal connections with patients can foster trust and understanding. The ability to effectively communicate with patients about their medication contributes to not only therapeutic relationships but also to positive patient outcomes. Helping patients regain their well-being provides a strong sense of purpose and accomplishment. Person-centred treatment engages with patients’ unique needs. Providing patients with information about their treatment can reassure them when they express concerns about medications. Pharmacists may clear up patients’ reservations about medications and offer reassurances about the potential side effects or outcomes:

*You’ve had that consultation with the patient that you know there’s a particular medication that they. You want them to try all the team wants them to try, but they’ve got reservations about it and you can go and clear those reservations up and give them reassurance that what they’re thinking might not be the case.*

*(P11)*

Figure 2 shows the theme diagram for Positive Aspects and Satisfaction.

#### 3.1.3. Theme 3: Challenges and Barriers

Mental health pharmacists face several challenges that can hinder the delivery of optimal care. These challenges include stigma and attitudes towards mental health, role ambiguity and interdisciplinary collaboration, educational and practice gaps, difficult patient interactions, and institutional challenges, which can influence continued success and satisfaction in this role.

##### Stigma and Attitudes Towards Mental Health

Despite a positive shift in public attitudes, stigma remains a pervasive challenge in mental healthcare. Mental health is considered *“still quite taboo,” (P1)* and may extend to their families, refusing to acknowledge the reality of their loved one’s auditory hallucinations, and attributing the behaviour to moral failings. Also, mental health patients fear being labelled and judged. The fear of judgement extends beyond the individual because the diagnosis of mental health might be seen as a personal failure. However, in one case, patients feel *“nothing wrong with them.” (P11)*

Another challenge is that some patients reflect on their previous experiences and avoid recognising their health conditions. As patients hide themselves *“behind shadows,” (P6)* they might miss access to the support they need.

Society may hold negative perceptions and fear surrounding mental health treatment. People may call mental health facilities by *“nasty names.” (P1)* Individuals with certain mental health diagnoses, such as schizophrenia or bipolar disorder, may be considered *“dangerous” (P2).* Even students or trainees feel *“frightened” (P11)* when working in mental health wards.

##### Role Ambiguity and Interdisciplinary Collaboration

Healthcare professionals may *“underrate” (P8)* mental health pharmacists in patient care

*I think as a healthcare provider, pharmacists sometimes are underrated. We don’t appreciate how much of an input we can have.*

*(P8)*

However, pharmacists may be fully integrated into the team’s decision-making processes and make great contributions to patient care. Moreover, communication and collaboration within multidisciplinary teams may be a challenge due to conflict in approaches to patient care. In some instances, teamwork is challenged by colleagues or by a lack of awareness between professions. Interviewed pharmacists reported that they navigated the challenges by highly valuing team spirit and providing evidence-based recommendations.

##### Educational and Practice Gaps

Current pharmacy education lacks an emphasis on mental health topics, leaving new pharmacists unprepared for the complexities in practice. Mental health medications are only briefly addressed in the training and take a small component of the general curriculum. Even mental health education was limited to reading case studies or notes, or on-the-job training. Even a few research participants were not aware of the potential for working in mental health until they started in the field. Additionally, unstructured clinical supervision, limited funding, and skill-mix staffing may reduce confidence in knowledge. Patients’ needs may disconnect from pharmacists’ knowledge and skills.

##### Difficult Patient Interactions

Patients’ mental uncertainty may impede the collection of necessary information for effective medications:

*Because obviously mental health patients, depending on their condition, vary very much at their level of engagement and the quality of the interaction. So it’s not just straightforward explaining something to somebody.*

*(P7)*

Pharmacists then have to determine the right time to engage with them. In cases, patients are in a state of relapse and unable to provide consent or engage in their care, reflecting an ethical dilemma. Managing patient expectations and dealing with conflicts is an aspect of complexity. Conflicts can arise with family members:

*You get conflicts with the carers or family and friends of the patients as well about what is the right thing.*

*(P2)*

Patients may also resist medications or fail to adhere to prescribed treatments. Relapsed patients may return due to side effects resulting from discontinued treatment. Research participants also reported the risks and emotional impact of working in mental health. From facing personal harassment in the workplace, to working alongside patients with a history of violence and aggression, to managing risks, pharmacists face a cumulative, overwhelming emotional burden.

##### Institutional Challenges

In the shortage of mental health pharmacists, other healthcare professionals struggle to perform their roles effectively, heavily burdening the existing team. Professional development and operational goals can be compromised, and the capacity to effectively use resources is constrained. A national agenda on mental health services cannot be achieved.

The complexity of systems makes it difficult to effectively manage time. One participant stated, *“Systems in place are quite convoluted. It’s quite difficult to know who to go to for what specific thing” (P2).*

Time pressures become a barrier because professionals struggle to complete tasks within tight timeframes. Additionally, work complexity reflects a lack of guidelines for treatment, and heavy workload challenges meeting high standards.

Prescribing unlicensed medications challenges engagement with mental health treatment. This issue is further complicated when patients or caregivers encounter difficulties in obtaining the appropriate medications, or when patients’ medications are not reviewed after their conditions improve. In some cases, new prescriber pharmacists are not capable of prescribing due to a lack of practice and management of adverse effects. Insufficient funding represents a consistent issue that hampers quality care. Additionally, while students of different disciplines receive low compensation for professional development, pharmacy students do not even receive any:

*My son being a physio student gets the £5000 a year learning support and gets travel expenses for placements. Physio, OT, and nursing students get that. Pharmacy doesn’t.*

*(P9)*

**Figure 3:**
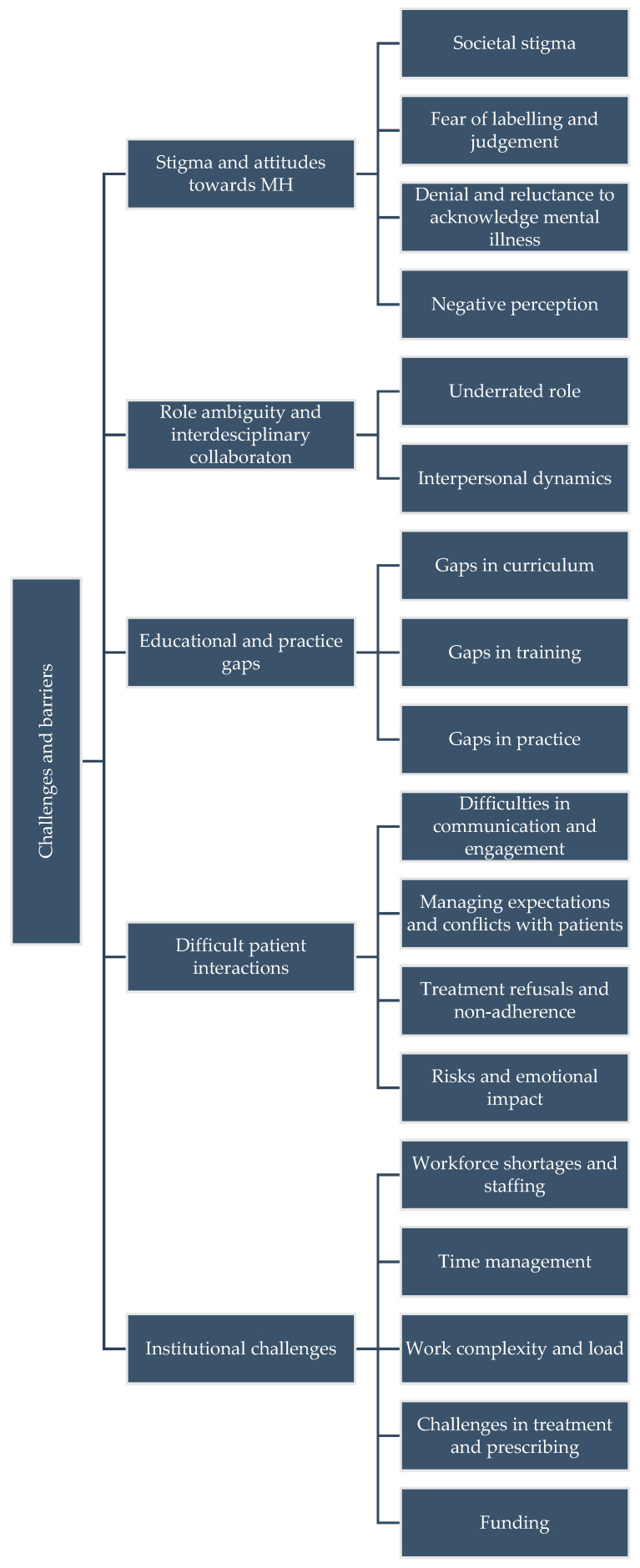
Theme diagram for Challenges and Barriers.

Figure 3 shows the thematic linkages.

#### 3.1.4. Theme 4: Views and Recommendations

##### Views About Supplementary Courses

Current training courses do not prepare pharmacists for prescribing in mental health settings. Specialised mental health training (or certification) should be incorporated into prescribing courses to benefit early-career pharmacists, enhancing their competencies. Bespoke training can focus on conditions commonly encountered in mental health settings. Additionally, the healthcare system lacks the capacity to fully integrate pharmacists into prescribing roles, while pharmacists lack confidence and opportunities to take on the prescribing responsibilities. In some cases, pharmacists’ tasks are not different from their previous ones (before the qualification), limiting the potential benefits of independent prescribing.

*I found that the prescribing course was heavily weighted to physical things, which are important, but there just wasn’t enough in there for me to then go back to my practice and say, ‘OK, well, I learned this.*

*(P10)*

*When you do that [postgraduate certification], then you kind of get to learn about a lot of different things within mental health. And then you can apply that to the get the resources, you get the knowledge.*

*(P3)*

##### Views About Prescribing for Pharmacists

Prescriber-ready pharmacists alleviate the burden on other healthcare professionals, enhancing healthcare delivery and treatment outcomes. Interviewees expected an increased role of prescribing pharmacists in the future due to healthcare demands. However, many research participants expressed concerns about the readiness and confidence to prescribe after MPharm studies:

*Some will not want to prescribe because they’re not confident; some are going to be overconfident and wanting to prescribe without any kind of experience in a particular area.*

*(P7)*

The expectations placed on newly qualified pharmacists may mismatch their actual experience and readiness for prescribing. Therefore, new graduates should obtain additional qualifications before prescribing. Interviewees also worry about the generalist nature of the MPharm qualification. While other healthcare professionals (generalist junior doctors, trainee GPs) have had an additional two or three years of training before taking on responsibilities, pharmacists have not. Additionally, the transition from university to prescribing was seen as a significant leap in responsibility for many interviewees. Mental health pharmacists need practice and exposure to real-world scenarios to develop their confidence.

##### Recommendations

Interviewees recommend increasing awareness about mental health to promote a more inclusive approach to mental health education and career development for pharmacists. These include (1) raising public and healthcare sector awareness to normalise mental health; (2) promoting community-based mental health initiatives to reduce the burden on hospital admissions; (3) increasing visibility of career opportunities in mental health pharmacy; and (4) enhancing educational exposure through events and job fairs.

Interviewees also recommended improving content in the university pharmacy curriculum, expanding lectures and modules *“to give a bit more insight into mental health” (P8),* and spanning across multiple years of study. However, a few participants insisted on introducing mental health modules in later years of study. Moreover, bringing in external experts may cultivate interest early on in mental health pharmacy education.

Interviewees requested more direct experience in mental health. Incorporating mental health into the MPharm curriculum could enhance its legitimacy, and workplace placements may foster deeper knowledge and confidence when dealing with mental health patients. In particular, curriculum coverage of mental health topics should be more in-depth and universal for learners, even if they do not intend to specialise in it.

Recommendations for structured support in mental health include (1) establishing clear entryways into the mental health field to make the transition easier for pharmacists; (2) implementing systematic mentorship programmes; (3) developing a team-based model on mental health wards; and (4) encouraging early discussions about mental health placements in education programmes.

**Figure 4:**
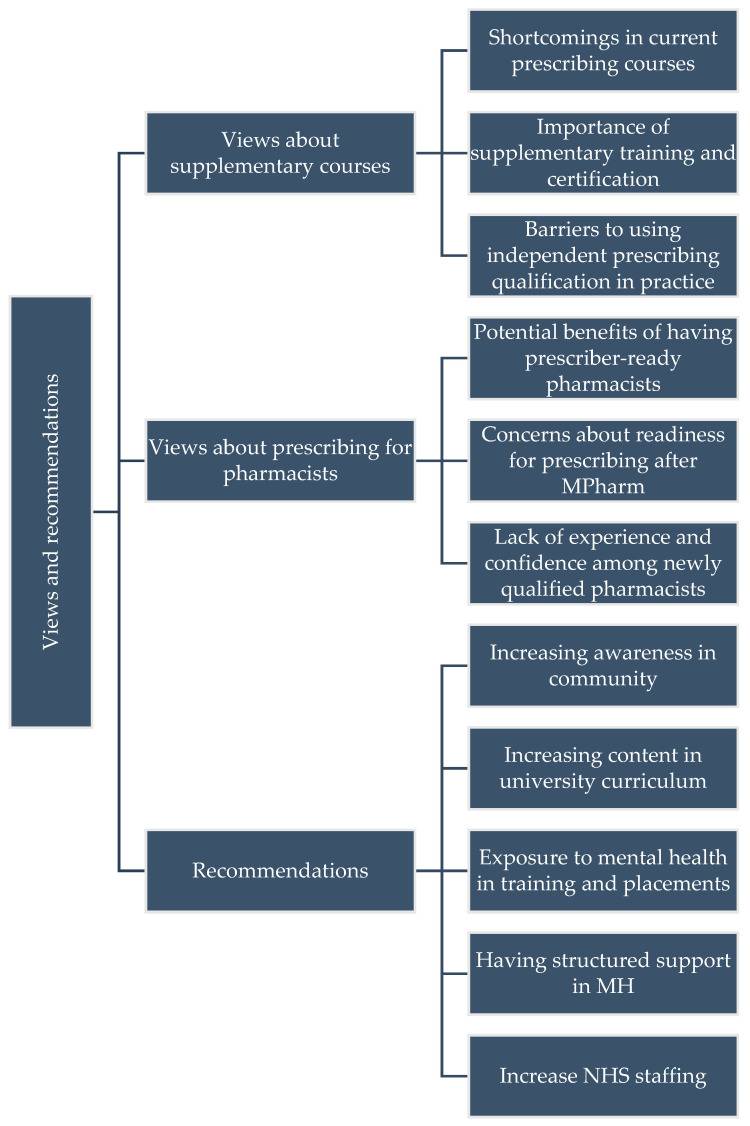
Theme diagram for Views and Recommendations.

An increase in NHS staffing responds to demands in mental healthcare. The increased staff-to-patient ratio would improve overall patient care and reduce the workload on individual healthcare professionals. Adding more pharmacists to mental health settings is needed to provide the necessary expertise and support. Newly qualified pharmacists should receive supportive supervision to adequately prepare them for a prescriber role (Figure 4).

#### 3.1.5. Interpretation and Semantic Linkages

**Figure 5:**
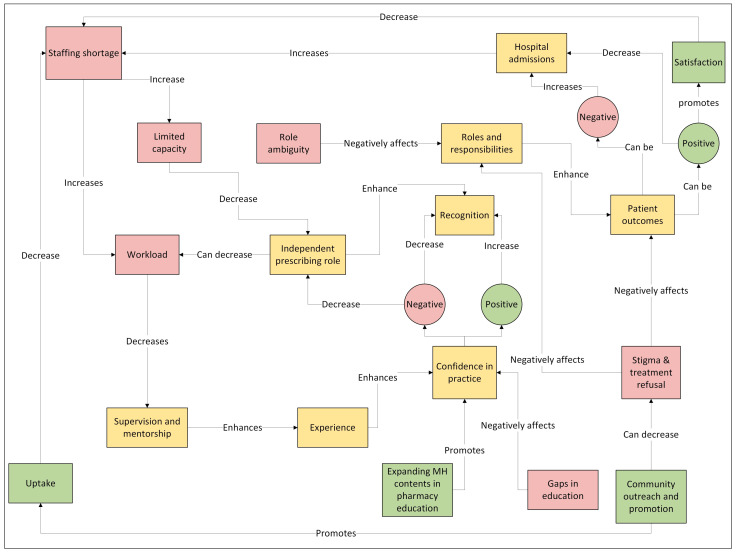
Semantic linkages among themes.

Semantic linkages were explored across the themes to identify and understand a deeper, structural, and systemic integration of pharmacists’ roles, experiences, and viewpoints (Figure 5). In this diagram, the colours and shapes represent the type of factor and its corresponding influence. Red boxes represent negative factors or barriers. Yellow boxes denote neutral or contextual factors. Green boxes highlight positive factors or enablers. Circular nodes represent outcomes, where a red circle reflects a negative outcome, and a green circle reflects a positive outcome. The arrows indicate the direction of influence: “increases” or “enhances” or “promotes” denote a positive or amplifying effect, while “decreases” or “negatively affects” denote a reducing or detrimental effect.

Pharmacists described the myriad roles and responsibilities they have within the service; however, they felt that their roles are still *underrated (P8)* and *under-evaluated (P9).* Despite these concerns, the positive impact on patients can compensate for such limitations, as satisfaction from positive patient outcomes carries an intrinsic value and contributes to the *overall satisfaction in the role (P1).* For some, it makes all the efforts *worthwhile (P7).* Stigma and illness denial result in non-adherence to MH treatment therapy by patients, which makes it harder for pharmacists to perform their patient-facing roles. There needs to be more community outreach initiatives and educational interventions that could reduce societal stigma among individuals and increase recognition of pharmacists in mental health. This could promote interest in others to join this field and would contribute to lower hospital admissions in the long run, thereby reducing the workload. This could also *reduce hospital admissions (P1, P8–10)*.

Gaps in the undergraduate curriculum and lack of experience result in *low preparedness* among newly qualified prescriber pharmacists and consequently, hinder their ability to *prescribe confidently (P5, 7–11).*

Those who already have an independent prescriber qualification often are *unable to utilise it* due to *staffing shortages* and *limited capacity* to take on this role *(P9, P10).* Increased *promotion* of mental health opportunities could improve the uptake *(P1, P8).* If pharmacists start prescribing independently, it may ease the burden on other healthcare professionals and *improve the service (P1).* This would also contribute to greater recognition of pharmacists generally and within multidisciplinary teams. *Expanding MH contents and placement opportunities* in the pharmacy curriculum would improve knowledge and confidence among new graduates *(P1, P9).* These prescriber-ready graduates could be supported through *supervision* and *mentorship* by *senior pharmacists.* This would give them *confidence* to prescribe; however, ongoing issues related to *staffing, workload, and time constraints* prevent them from assuming these roles *(P3, P9, P10).*

## 4. Discussion

The findings highlighted key roles pharmacists perform in secondary-care NHS Trusts that included clinical, operational, and strategic roles. Previous studies have highlighted pharmacists’ contributions to medicines management [21], counselling and patient consultations [17,22], education [20], managing supplies, prescribing [18,19], and supporting specialised services such as the clozapine service [27]. However, findings also revealed that there is a sense of concern regarding the underrated roles and undervalued contributions pharmacists make in this area. In the Royal Pharmaceutical Society (RPS) report [12], it was mentioned that the public at large who suffer from mental health issues are still unaware of the benefits of pharmacists or pharmacy services in their treatment and medication management. The report mentioned that there is a need for pharmacy professionals to take the lead and work with service users, healthcare professionals, charities, and other stakeholders to raise awareness of their roles and the value they bring to mental health services. Further engagement is needed among specialist mental health services, general practices, and community pharmacies. The report identifies the disconnect among these care levels in the NHS. This engagement and its potential benefits need to be explored in the future.

Taylor et al. (2011) evaluated pharmacist contribution to clozapine services [27], highlighting their impact on the service, which was largely positive; however, the contribution of the pharmacist in the service was mainly as a support and largely undefined. This means that although pharmacists play an important role in improving the service from an organisational, societal, and economic perspective, their role or the value they bring to the team still needs to be clarified. There is a potential for more clarification on the individual roles pharmacists are expected to perform apart from supporting other healthcare professionals in the team when it comes to advanced roles, such as specialised clinics. An audit conducted in the NHS has identified further areas for improvement in this service and has reported the proactive roles pharmacists have performed in executing the service [28]. This would promote visibility, recognition, and motivation to work/join this field that could help in the face of the current funding and staffing shortages plaguing the NHS.

A challenge that was identified by the participants was the education and training gaps. Participants seemed sceptical about the prescribing readiness of the newly graduated pharmacists and expressed the need for further training and gaining experience before prescribing MH medications. This is echoed in the RPS report [12] that the knowledge and expertise of pharmacists vary across places, and not all pharmacists will have confidence in treating patients with mental health conditions. It was highlighted that the mental health content in the university’s MPharm curricula may not be enough on its own to empower graduates in prescribing such medications. Gorton and colleagues reported that the MH education in the MPharm is theoretical and knowledge-based rather than applied [29]. In a study by Johnson & Earle-Payne [30], pharmacy staff in general practice in Scotland identified a need for further training in several areas of mental healthcare, ranging from general ones such as sign and symptoms and monitoring to specialised areas such as assessing suicide and self-harm risk, biopsychosocial assessment, and reviews for antidepressants, benzodiazepines, valproate, and other psychotropic medications. Such trainings were believed to aid them in better supporting patients with MH conditions. The RPS report calls on the institutions associated with mental health teaching, training to ensure that pharmacists are empowered in their knowledge and practice to support this population [12]. Currently, research on identifying knowledge gaps on MH in the MPharm curricula of HEIs in the UK is limited. This requires a large-scale consultation with all stakeholders to map the current teaching delivered on the topic in the MPharm programme and identify commonalities and recommended additions. Similarly, research is needed to uncover the gaps in the practice of pharmacists in MH to identify areas where additional CPD learning could be designed and delivered. This study has highlighted that UK pharmacists perceive that there is a knowledge gap; however, further in-depth research is needed to identify and address those gaps.

One of the ways to address the knowledge gap in the short-term is to involve specialists pharmacists. Specialist pharmacists could be involved in the psychiatric liaison team (PLT) in primary- and secondary-care settings. An example is the PLT in Sunderland Royal Hospital. Patients with a psychiatric diagnosis and a comorbidity of physical illness are reviewed by PLTs to ensure proper treatment plans are devised to treat mental illness and physical illness together. This is an example of advancing pharmacists’ roles and widening participation. This would also contribute to their recognition both within the Trusts and in society [12].

Participants highlighted that stigma and treatment denial are barriers to treatment and result in non-adherence to treatment. This results in poor treatment outcomes and increased hospital admissions. Additionally, 50% of the patients who take antidepressants stop adhering to their treatment after one year [31]. Stigma has been reported previously, and several interventions have been designed and implemented to address societal and anticipated stigma perceived by patients with MH. Recent evidence from the UK reports that it has been somewhat reduced; however, it remains relevant. Mental health stigma continues to be a barrier to accessing mental healthcare among ethnic minorities in the UK.

There is a dearth of literature that reports stigma among ethnic minorities in the UK, and further research is needed to address the root causes of stigma. Research that documents the lived experiences of individuals and identifies the sociological process that transforms a lived experience into a stigma perception is required. Such research would highlight the gaps in existing interventions and facilitate the design of effective strategies to tackle this issue. In addition, studies have reported that healthcare providers mentioned the absence of stigma on their part; however, this has not been independently investigated or validated by service users to determine if they echo this narrative.

Evidence also highlights that people with MH who are from ethnic minority groups may have lower treatment engagement [32]. Memon and colleagues identified several factors that impact treatment engagement in this population. These include personal, environmental, social, cultural, and organisational factors. The study provided several recommendations, such as increasing mental health literacy, mental health awareness, campaigns to tackle stigma, and training healthcare professionals on delivering culturally sensitive care [33]. Further studies are needed to understand how pharmacists can improve treatment engagement in ethnic minorities.

Pharmacists are involved in counselling patients and educating them about how to take their medications. The New Medicines Service (NMS), which is now a part of the community pharmacist role, has been reported to increase medication adherence [34]. However, NMS and medication utilisation reviews (MUR) are not funded for mental health services in the community pharmacies. Hence, this service may not be utilised for addressing patients’ medication needs in mental health specifically. This could be an area for further exploration.

The NHS mental healthcare service is fragmented, and there is a need to provide community pharmacies with access to medical records and have more GP-based pharmacists to improve the MH service. Since this work focused on pharmacists involved in secondary care, it is recommended to conduct studies in primary care settings to evaluate pharmacists’ roles and responsibilities and the impact they have on patient care. The practice in primary care, coupled with these findings, would provide a clear picture of the roles pharmacists perform in all NHS settings, identify gaps, and highlight new research avenues. This study was not without limitations.

Potential limitations are a limited sample size from sites, and with qualitative data, it might limit the transferability of findings to other trusts, as working patterns among Trusts, although assumed to be largely similar, may still have unique aspects owing to the differences in geographic location, capacity, clinical burden, and resources. There was an element of sampling bias, as only those who were more engaged/motivated about MH services may have participated. Additionally, some investigators were also employed by the Trusts and contributed to the recruitment of participants. This meant that there may be a relationship between the researcher and the participant. However, the interviews were arranged and conducted by other investigators who did not have a relationship with the participants. Anonymity was ensured, and therefore, the investigators who may have had a relationship with participants could not know if the participants were interviewed. The interviews were coded, and themes were reviewed by investigators who were pharmacists, and therefore, this could have brought an element of bias in the analysis. To minimise this bias, an investigator who is a qualitative researcher and a non-pharmacist, and was based outside the UK, was involved in the independent coding with the other investigators. Triangulating the interpretations by investigators helped eliminate the bias and ensured that the findings were valid. Lastly, data saturation was not achieved in the interviews, and the themes may not be fully developed from the findings. Nonetheless, this study has highlighted some of the viewpoints UK pharmacists have regarding their role in MH service. It may serve as a primary source of information and provide a direction in designing future studies. Further research involving a larger sample size with geographic diversity is recommended.

## 5. Conclusions

Pharmacists in the NHS secondary-care settings perform a wide variety of roles, which were reported previously in the literature. The gaps mostly identified in this work have been reported previously in the UK clinical settings, suggesting that the challenges pharmacists face in their roles in mental health persist over time. A key novel finding of this study was the perceptions of pharmacists regarding the newly qualified prescriber-pharmacists. Although newly qualified prescriber-ready pharmacists provide hope of replenishing the NHS’s severely depleted workforce, there are concerns about their efficiency in the short term, and they may not be as prescriber-ready as expected. This work has highlighted the current roles of pharmacists in mental health in secondary care settings in the NHS, and additional findings from pharmacists in primary care settings who deal with individuals with mental health conditions, and with minority ethnic populations with mental health conditions, would provide a collective account of the current roles of pharmacists and their potential. Further research is needed to uncover challenges at the primary care level to grasp the full understanding of the challenges the pharmacists in the NHS face regarding mental healthcare, especially at a time when the UK continues to become more diverse.

## Figures and Tables

**Table 1:** Participant information (*n* = 11).

Participant Information	Sample Count (*n*)	Percentage (%)
**Gender identity**		
Man	2	18.2
Woman	9	81.8
**Age group**		
Less than 30 years	3	27.3
Between 31 and 40 years	3	27.3
Between 41 and 50 years	2	18.2
Between 51 and 60 years	2	18.2
Above 60 years	1	9.1
**Highest level of Pharmacy-related education and training**		
MPharm	4	36.4
Postgraduate Diploma	2	18.2
Independent prescriber	4	36.4
Postgraduate MSc	1	9.1
Workplace *		
PPH at BHNHSFT	5	45.5
BSMHFT	6	54.5
**Average weekly working hours**		
Between 8 and 24 h	2	18.2
Between 25 and 40 h	8	72.7
More than 40 h	1	9.1
**Working at another setting**		
No, I do not work in any other healthcare setting	9	81.8
Yes, I also work in another healthcare setting	2	18.2
**Work experience in the UK**		
Less than 5 years	2	18.2
Between 5 and 10 years	1	9.1
Between 11 and 15 years	3	27.3
Between 16 and 20 years	1	9.1
More than 20 years	4	36.4
**Training or continuing education programmes in mental health in last 5 years**		
No	4	36.4
Yes	7	63.6
**Time spent on training and/or continuing education programmes in last 5 years**		
0–30 h	1	9.1
31–60 h	1	9.1
More than 60 h	5	45.5
Not applicable (if the option ‘No’ was selected in previous question)	4	36.4

* PPH = Prospect Park Hospital; BHNHSFT = Berkshire Healthcare NHS Foundation Trust; BSMHFT = Birmingham and Solihull Mental Health NHS Foundation Trust.

## Data Availability

The original contributions presented in this study are included in the article/Supplementary Materials. Further inquiries can be directed to the corresponding author.

## Supplementary Materials

The following supporting information can be downloaded at: https://www.mdpi.com/article/10.3390/healthcare13202602/s1, S1: Demographic form and interview guide; S2: Codebook; S3: Thematic analysis document; S4: Quantitative raw dataset.
