# Supplementary material for: Exploring Pharmacists’ Perceptions of Their Current Role in Mental Health Trusts in England: A Qualitative Study
Source: Healthcare (Basel). 2025 Oct 16;13(20):2602. doi: 10.3390/healthcare13202602 (PMC12563904; doi:10.3390/healthcare13202602)
Supplement: Supplementary file 1 [file healthcare-13-02602-s001.zip › S1.Demographic form and interview guide.pdf]

Demographic form

PHARMACIST INFORMATION

1. **Please indicate your age group.**
   - o Less than 30 years
   - o Between 31 to 40 years
   - o Between 41 to 60 years
   - o Between 51 to 60 years
   - o Above 60 years
2. **Which of the following best describes you?**
   - o Man
   - o Woman
   - o Prefer to self-describe
   - o Prefer not to say
3. **Please indicate your highest level of Pharmacy-related education and training.**
   - o MPharm Pharmacy
   - o Pharm.D
   - o Residency trained
   - o Fellowship trained
   - o Master of Pharmacy (MSc)
   - o PhD
4. **How many hours do you work on average on a weekly basis at the hospital?**
   - o Less than 8 hours
   - o Between 8 - 24 hours
   - o Between 25- 40 hours
   - o More than 40 hours
5. **In addition to the primary setting, do you also work in any other healthcare setting?**
   - o No I do not work in any other healthcare setting
   - o Yes I also work in another healthcare setting
6. **If you selected Other, please specify:____________________**

7. **How many years have you worked as a pharmacist in The UK?**
   - o Less than 5 years
   - o Between 5 – 10 years
   - o Between 11 – 15 years
   - o Between 16 – 20 years
   - o More than 20 years

ALL THE FOLLOWING QUESTIONS WILL REFER TO YOUR INVOLVEMENT AT THE COMMUNITY PHARMACY SETTING:

8. In a typical week, how much time of your practice is dedicated to the mental health service?

- o Less than 25% of the time
- o Between 25% – 50% of the time
- o More than 50% of the time

9. In the last 5 years, have you completed any formal training or continuing education programs in mental health?

- o No
- o Yes I have completed 'Other' formal trainings and continuing education programs

10. If you selected Other, please specify:____________________

11. How much time in the last 5 years in total did you spend in these training or continuing education programs?

- o 0 – 30 hours
- o 31 - 60 hours
- o More than 60 hours
- o Not applicable (if you selected the option 'No' in previous question)*

CONSENT FOR INTERVIEW

Would you like to participate in the interview?

- o Yes
- o No

If you indicated that you are interested in participating in the interview, please provide your email address and 2-3 time slots for your availability below. Please note that you will be contacted on the email provided for arranging an interview on Microsoft Teams.

___________________________ (open text)

Thanks for your participation!

If you felt that answering questions caused some anxiety or distress to you. The NHS has introduced a confidential text support service, you can access support by texting FRONTLINE to 85258 for support 24/7. This service is available to all our NHS colleagues who have had a tough day, who are feeling worried or overwhelmed, or who have a lot on their mind and need to talk it through.

-End of survey-

Interview guide

Thank you for taking part in this interview, I really do appreciate the time you have given. Before we begin, I want to make it clear that if you wish to skip any question(s) during the interview, or if you want to stop the interview, all you have to do is say; you do not need to give any explanation for doing so.

Are you happy for me to begin?

Section 1

1. Can you tell me a bit about yourself? For example, could you please tell me about your work setting, current role?
2. What do pharmacists do for patients with mental health?

   Prompt: Can you describe what a typical day at work within mental health service involves for you?

Section 2

The next few questions are going to be regarding your feelings, attitudes, perceptions regarding mental health service.

3. What do you think about the work you are doing in mental health service? How do you feel about the work you do?
4. – what are the challenges?
5. what we the positives?

   Prompt: Can you describe to me any practical issues/concerns (if any) you find with your work?

   Prompt: Are there any particular patient scenarios/experiences you want share with us, that demonstrates a positive or negative aspect of your role?

Section 3

The next few questions are going to be regarding how good and bad you feel about the work of pharmacists in mental health.

6. Being in a hospital, are you aware of the NHS/GPhC guidance and responsibilities for pharmacists working in mental health services?
7. How do you feel you are contributing/ accomplishing around mental health considering the guidance and responsibilities delegated by the NHS/GPhC?

   Prompt: What are the barriers/challenges you face during practice in mental health service?

8. Please share your expertise on how these challenges could be overcome.

   Prompt: When you look at yourself, do you feel that you notice any barriers or stigma towards mental health service from your or patients' perspective?

   Prompt: Please feel free to share more about your experience (if you like).

Section 4

The next few questions are going to be regarding your thoughts on the gaps in field of mental health and your recommendations for the role of pharmacists in this area.

9. What do you think are the gaps in training/practice pertaining to the effective mental health service provided by pharmacist?

   Prompt: Have you completed any certification or training in mental health and how does that help in this role?

   Prompt: Given the current push towards independent prescribing for pharmacists, do you have any specific recommendations for mental health pharmacists?

   Prompt: Do you have any recommendations for future pharmacists who are studying in MPharm at the university?

   OR If you could change something about mental health care, what would it be?

Thank you! Is there anything which we did not cover in the questions? Please let us know.

If you felt that answering questions caused some anxiety or distress to you. The NHS has introduced a confidential text support service, you can access support by texting FRONTLINE to 85258 for support 24/7. This service is available to all our NHS colleagues who have had a tough day, who are feeling worried or overwhelmed, or who have a lot on their mind and need to talk it through.

Thanks! This is the end of the interview. I will stop the recording.

Thank you and have a nice day!

End of interview
