# Supplementary material for: Exploring Pharmacists’ Perceptions of Their Current Role in Mental Health Trusts in England: A Qualitative Study
Source: Healthcare (Basel). 2025 Oct 16;13(20):2602. doi: 10.3390/healthcare13202602 (PMC12563904; doi:10.3390/healthcare13202602)
Supplement: Supplementary file 1 [file healthcare-13-02602-s001.zip › S2.Code book.pdf]

Codebook

Project title:

Code	Definition
**Theme 1: Role and responsibilities**	
**Minor theme 1.1. Medication management**	
Medication information and advice	Pharmacists respond to request/queries from clinicians, healthcare professionals, patients for pharmaceutical information and advice.
Medication reconciliation	Pharmacists reconcile medications prescribed for by clinicians. Checking dose, documenting medications, advising on side effects.
Medication review and optimisation	Pharmacists ensure optimizing effects of drugs on patients. Checking for drugs' appropriateness, safety, and contraindications or interactions.
Monitoring and assessing compliance	Pharmacists ensure and report patients' compliance and progress. See patients and suggest blood/urine test.
Support in specialised services	Pharmacists advise and ensure safety and effectiveness of outpatient prescriptions including clinic (dementia, pain, mental health).
**Minor theme 1.2. Clinical involvement**	
Involvement in Multidisciplinary Team Meetings (MDTs), ad-hoc meetings, and pharmacy meetings	Pharmacists engage in series of events/meetings to advise on medication issues/concerns and provide recommendations for treatment.
Consultations and counselling	Pharmacists provide consultations and counselling on medications
Documentation and follow-up	Pharmacists document and follow up drug treatments, such as writing patients' notes
**Minor theme 1.3. Operational duties**	
Operational and dispensary support	Pharmacists dispense drugs to patients
Managing supplies	Pharmacists perform logistics to ensure adequate supply of drugs
Discharge medication planning	Pharmacists ensure and prepare patients for safe discharge
**Minor theme 1.4. Involvement in teaching, research, and quality assurance**	
Audits and quality assurance	Pharmacists perform periodical and overall auditing of drug prescriptions.
Research and information support	Pharmacists generate knowledge through research/trial-related activities and provide evidence-based recommendations.

Teaching, preceptorship and education	Pharmacists engage in teaching, training, and experience exchange with colleagues and coming generations.
**Minor theme 1.5. Leadership and strategic roles**	
Leadership roles	Pharmacists organize and coordinate teams
Policy development and implementation	Pharmacists engage in policy processes on various issues relating to drugs and treatment (compliance, guidelines, policy development, governance)
**Theme 2: Positive aspects and satisfaction**	
**Minor theme 2.1. Satisfaction**	
Satisfaction and recognition in job	Pharmacists' satisfaction at workplace (e.g., feeling rewarding, effectiveness) as shown by pharmacists and by team members
Satisfaction with team and work environment	Factors (e.g., shared values, team spirits, work atmosphere) that contribute to pharmacists' satisfaction at workplace
Peer learning	Pharmacists describe/reflect on team's learning/cooperation.
**Minor theme 2.2. Recognition of mental health and pharmacy services**	
Recognition within team	How pharmacist contribution to team is recognized
Change in public attitude towards mental health	Mental health is now more openly accepted and discussed
**Minor theme 2.3. Positive impact on patient**	
Building relationships with patients	How pharmacists build relationships with patients (e.g., talk, build rapport)
Witnessing patient progress	Pharmacist reflection (e.g., emotions, thoughts) on patient progress.
Promoting person-centred care and reassuring patients	Pharmacist reflection (e.g., emotions, thoughts, actions) on person centred care
**Theme 3: Challenges and barriers**	
**Minor theme 3.1. Stigma and attitudes towards mental health**	
Societal stigma	Pharmacist perspective (e.g., negative, positive, description, reflection) on stigma against mental
Fear of labelling and judgment	The fear of being labelled and judged
Denial and reluctance to acknowledge mental illness	How people admit (e.g., accept, negate, deny) mental illnesses

Negative perception	Negative perception (or feel, or reflection) of people's mental
**Minor theme 3.2. Role ambiguity and interdisciplinary collaboration**	
Underrated role	Confirmation that pharmacists' role in teams is not fully understood.
Interpersonal dynamics	The multi-dimensional relationship pharmacists perform in a team
**Minor theme 3.3. Educational and practice gaps**	
Gaps in curriculum	Pharmacists are not trained in official curriculum mental-health related skills/competencies
Gaps in training	Pharmacists lack further professional training in mental-health related skills/competencies
**Minor theme 3.4. Difficult patient interactions**	
Difficulties in communication and engagement	Pharmacists describe their difficulties in communication and engagement with people with mental health problems
Managing expectations and conflicts with patients	Pharmacists describe their difficulties in managing expectations and conflicts with patients with mental health problems
Treatment refusals and non-adherence	Pharmacists' reflection on treatment refusals and non-adherence among people with mental health conditions.
Risks and emotional impact	Pharmacists' reflection on risk and emotions working with people with mental health problems
**Minor theme 3.5. Institutional challenges**	
Workforce shortages and staffing	Challenges in staffing (quality, quality) in mental health at pharmacies. Their impacts at work.
Time management	Lack of time management (time allocation, skills)
Work complexity and load	Pharmacists' reflection on the complexity at work, and workload that influence mental health issues
Challenges in treatment and prescribing	Challenges (reluctance, underestimate) in treatment and prescribing for people with mental health problems
Funding	Lack of funding (allocation, management) for mental health care at pharmacies
**Theme 4: Views and recommendations**	
**Minor theme 4.1. Views about supplementary courses**	
Shortcomings in current prescribing courses	Shortcomings in current prescribing courses on mental health for trained pharmacists
Importance of supplementary training and certification	Views (areas of training, specialized courses, certificating) on importance of supplementary training and certification

Barriers to using independent prescribing qualification in practice	Capacity, people resource, day-to-day work practice that inhibit using independent prescribing qualification in practice
**Minor theme 4.2. Views about prescribing for pharmacists**	
Potential benefits of having prescriber-ready pharmacists	Potential benefits of having prescriber-ready pharmacists
Concerns about readiness for prescribing after MPharm	Concerns about readiness for prescribing after MPharm
Lack of experience and confidence among newly qualified pharmacists	Lack of experience and confidence among newly qualified pharmacists
**Minor theme 4.3. Recommendations**	
Increasing awareness in the community	Reflection (scope, advantages, disadvantages) on increasing awareness about mental health in the community
Increasing content in university curriculum	Reflection (lecture, module training, interest) on increasing content in university curriculum
Exposure to mental health in training and placements	Exposure (placement, experiential training, interest enhancing) to mental health in training and placements
Having structured support in mental health	Various support for mental health training (multiple entry points, mentorship, exchange)
Increase in NHS staffing	Increase in NHS staffing (quantity, quality, funding)
