# Supplementary material for: Exploring Pharmacists’ Perceptions of Their Current Role in Mental Health Trusts in England: A Qualitative Study
Source: Healthcare (Basel). 2025 Oct 16;13(20):2602. doi: 10.3390/healthcare13202602 (PMC12563904; doi:10.3390/healthcare13202602)
Supplement: Supplementary file 1 [file healthcare-13-02602-s001.zip › S3.Thematic analysis document.pdf]

Qualitative analysis

Table of Contents

Theme 1: Role and responsibilities 4
Minor theme 1.1. Medication management 4
Code: Medication information and advice 4
Code: Medication reconciliation 4
Code: Medication review and optimisation 4
Code: Monitoring and assessing compliance 5
Code: Support in specialised services 5
Minor theme 1.2. Clinical involvement 6
Code: Involvement in Multidisciplinary Team Meetings (MDTs), ad-hoc meetings, and pharmacy meetings 6
Code: Consultations and counselling 6
Code: Documentation and follow-up 7
Minor theme 1.3. Operational duties 7
Code: Operational and dispensary support 7
Code: Managing supplies 7
Code: Discharge medication planning 8
Minor theme 1.4. Involvement in teaching, research, and quality assurance 8
Code: Audits and quality assurance 8
Code: Research and information support 8
Code: Teaching, preceptorship and education 8
Minor theme 1.5. Leadership and strategic roles 9
Code: Leadership roles 9
Code: Policy development and implementation 9
Theme 2: Positive aspects and satisfaction 10
Minor theme 2.1. Satisfaction 10
Code: Satisfaction and recognition in job 10
Code: Satisfaction with team and work environment 10
Code: Peer learning 10
Minor theme 2.2. Recognition of mental health and pharmacy services 11
Code: Recognition within team 11
Code: Change in public attitude towards mental health 11
Minor theme 2.3. Positive impact on patient 11
Code: Building relationships with patients 11

Code: Witnessing patient progress 11

Code: Promoting person-centred care and reassuring patients 12

Theme 3: Challenges and barriers 13

Minor theme 3.1. Stigma and attitudes towards mental health 13

Codes: Societal Stigma 13

Code: Fear of labelling and judgment 13

Code: Denial and reluctance to acknowledge mental illness 13

Code: Negative perception 14

Minor theme 3.2. Role ambiguity and interdisciplinary collaboration 14

Code: Underrated role 14

Code: Interpersonal dynamics 14

Minor theme 3.3. Educational and practice gaps 15

Code: Gaps in curriculum 15

Code: Gaps in training 15

Code: Gaps in practice 15

Minor theme 3.4. Difficult patient interactions 15

Code: Difficulties in communication and engagement 15

Code: Managing expectations and conflicts with patients 16

Code: Treatment refusals and non-adherence 16

Code: Risks and emotional impact 16

Minor theme 3.5. Institutional challenges 17

Code: Workforce shortages and staffing 17

Code: Time management 17

Code: Work complexity and load 17

Code: Challenges in treatment and prescribing 18

Code: Funding 18

Theme 4: Views and recommendations 18

Sub-theme 4.1. Views about supplementary courses 18

Code: Shortcomings in current prescribing courses 18

Code: Importance of supplementary training and certification 19

Code: Barriers to using independent prescribing qualification in practice 19

Minor theme 4.2. Views about prescribing for pharmacists 19

Code: Potential benefits of having prescriber-ready pharmacists 19

Code: Concerns about readiness for prescribing after MPharm 20

Code: Lack of experience and confidence among newly qualified pharmacists 20

Minor theme 4.3. Recommendations 20
Code: Increasing awareness in the community 20
Code: Increasing content in university curriculum 21
Code: Exposure to mental health in training and placements 21
Code: Having structured support in mental health 22
Code: Increase in NHS staffing 22

Theme 1: Role and responsibilities

Minor theme 1.1. Medication management

Code: Medication information and advice

1. "I kind of help the medics if they have any queries..." P1
2. "I previously also worked in our medical information service." P2
3. "I would answer queries from consultants in the public of our outpatient services regarding specific medication choices in patients with complex physical and mental health backgrounds." P2
4. "We've got wards ringing us, and then we've got our patients and queries from the dispensary." P3
5. "Clinical query that might arise from at the Community mental health team and regarding their services that we provide." P5
6. "I respond to queries like how to switch from antidepressant A to antidepressant B." P5
7. "So providing medicines and advice for clinicians and patients and we run two pharmacies, we have a community." P7
8. "So I feel that we're a great advocate for appropriate treatment." P9

Code: Medication reconciliation

1. "If there's a new admission, I do like a Med rec... ensuring if it is appropriate, has been documented, compare that with tests like ECG, blood test, and vitals." P1
2. "Checking the medication they came in is quite important because oftentimes they would get meddled up with their medication or they're on an abundance of medication." P2
3. "If a new patient is admitted, we do medicine reconciliation and ensure compliance...I try and do MedRec within 24 to 48 hours when I see the patient." P4
4. "I will carry on an emergency medicine reconciliation... and document that." P5
5. "So anything regarding medication, I am interactions patients' prescription that could include reducing polypharmacy, medicine reconciliation, patient counselling, discussion with the patient for a new medication, reviewing side effects." P8
6. "I'll go on to clinically checking people's drug charts. You know any medicines that have been newly prescribed?" P10
7. "All the prescriptions are clinically checked and they are appropriate for the patients and then getting that ready and sending it back out to the wards and teams." P11
8. "We do medicines reconciliation, all of those bits anyway."P11

Code: Medication review and optimisation

1. "I would aim to review all of the patient's charts to see if they are taking their medication because compliance is an issue." P2
2. "My daily sort of job is to go through patients charts and through their medical notes and ensure that the antipsychotic antidepressant their psychotropic medications are safe to use, are optimum dosing." P5
3. "I will be reviewing patient charts, seeing their progress." P5
4. "If the side effects are too great for them, then I could speak to the consultant to get it altered." P6

5. “I'm responsible for running what we call an insight report which is a basic report which highlights all the high levels above 600 micrograms litre per every week and then we have to report those back to the clinicians directly and also document everything in the patient notes.” P7
6. "If patient requires switch from a medication to another medication either because patient wishes to change to something else or the side effect profile is not there is not something that patient can tolerate and then we help with the consultant to come be the alternative plan." P8
7. “Half day a week undertaking other clinical work for the teams. Medicines, information, enquiries, drug histories.” P9
8. "Just checking them for appropriateness, for safety, and in line with any sort of like, you know, contraindications or comorbidities or interactions etcetera." P10
9. "So I actually try and focus more on deprescribing than I do on prescribing." P10

Code: Monitoring and assessing compliance

1. "Ensuring any of their PRNs, especially sedatives and antipsychotics, are within range acceptable being reviewed." P2
2. "We have to assess their compliance to the medications and sometimes it requires seeing each patient a few times throughout their stay." P3
3. "Sometimes we have to do monitoring for them as well, like physical health monitoring as part of your treatment agenda." P3
4. "We check the patients are monitored correctly and any side effects are managed…If a patient has compliance issues, I speak to them to address these concerns." P4
5. "To see if there are any interactions... between the medication or indirect interaction." P5
6. "Monitoring requirement… if somebody is on lithium, there are certain requirement and monitoring is required that we have to prompt our team to do the regular blood test." P8
7. "Monitoring for new prescriptions and making sure that new prescriptions are clinically checked, professionally checked and appropriately ordered." P9
8. "Obviously see the patient, see how they're progressing, think about whether they need a change in doses, things like that." P11

Code: Support in specialised services

1. “we do outpatient prescriptions for melatonin for cams. We do memory clinic prescriptions…Depots, outpatient prescription of depots…” P1
2. “I also help out with our outpatient streams which include memory clinic which is dementia and also help out with the pain clinic and we also supply medication crisis team as well as our children ADHD services where who require melatonin." P2
3. “I'm also the clozapine pharmacist, so the lead for that, which is obviously one of the mental health medications with sort of a lot of monitoring associated with it.” P7
4. "Every day we're dealing with clozapine, which is, as you are probably aware of, a very high-risk drug if it's not properly monitored." P7
5. “If somebody is on lithium, there are certain requirement and monitoring is required that we have to prompt our team to do the regular blood test.” P8
6. "I also spend some time working for a psychiatric intensive care unit as their ward pharmacist." P10

7. "I spend some time in a week going to care homes...I go to homes where people often have dementia or, you know, other kind of underlying serious mental illness." P10
8. "I work in the acute setting, so I cover a psychiatric intensive care unit, Mental health unit." P11

Minor theme 1.2. Clinical involvement

Code: Involvement in Multidisciplinary Team Meetings (MDTs), ad-hoc meetings, and pharmacy meetings

1. "I take part in MDTS which involve like the medics, the nurses myself and kind of have a healthcare professionals and. Kind of other people that are involved in the care of the patient. And so have I attend those once a week. So we go through each patient..." P1
2. "We go through each patient... I'll raise them if I have concerns, but I also raise them during the week." P1
3. "It's a meeting...with the consultants, the doctors, social workers...physios and OTs as well as the nurses and any healthcare assistants." P2
4. "I also attend multidisciplinary team meetings which happen once a week. There are also other ad hoc meetings that are set up by the MDT, the multidisciplinary team, throughout the week." P3
5. "We also attend a lot of internal pharmacy meetings. For example, safety meetings or huddles, quality improvement huddles and things like that." P3
6. "We normally attend the MDT... where we collaborate with other healthcare professionals like medics, consultants, and others." P4
7. "We review the guidelines and give recommendations as a team…Discussing treatment plans for patients, monitoring progress, and making recommendations with references." P4
8. "I'm involved in the multidisciplinary team meeting." P5
9. "We have what we call multiple disciplinary team meetings where everyone's involved...we put our heads together." P6
10. "One of my wards has a meet and MDT every day, which obviously we can't do every day and the other is twice a week." P7
11. "Part of my role is to be part of MDT, which is multidisciplinary team." P8
12. "So from a clinical perspective, I attend to MDT meetings a week on our two men's ICU wards." P9
13. "Discussions with (staff) about uh second opinion approved doctors to consider what, um, whether somebody's treatment plan is appropriate." P9
14. "I attend their MDT meetings when I can." P10
15. "In the MDTs, I advised doctors on medication issues. Issues any queries they've got and I make recommendations for medications." P11

Code: Consultations and counselling

1. "Sometimes also consultations to speak to patients about any adverse reactions, treatment choice, and explanations of their new medications, especially for high-risk medications like clozapine and lithium." P2
2. "We offer to see patients on the wards. We give one-to-one sessions." P3

3. "A lot of this or what they call psychoeducation... explaining about the medications and also explaining to them [patients] about their illness." P3
4. "Counsel patients on medication side effects, importance of adherence, and what to do if they experience issues." P4
5. "I counsel the service users on what's going on with their medication and what the future plans are." P10
6. "Usually I'll speak to, you know, a few of the service users on the ward, just say hi to them, ask if they've got any questions about their medicines. I'll use things like choice and medication leaflets to just frame discussions." P10
7. "I'll go and speak to the patients."P11

Code: Documentation and follow-up

1. "I write notes for all of the patients." P2
2. "We call and check that medications are ordered and patients are taking them continuously…We contact community mental health teams to make sure plans are followed." P4
3. "It's not always about medication. Medication plays a part, but it's the aftercare, the talking, the therapy, etc." P6

Minor theme 1.3. Operational duties

Code: Operational and dispensary support

1. "also kind of support in the dispensary as well…if they need kind of like a pharmacist to come in and do some check in or something like that…" P1
2. "I also help the pharmacy team" P1
3. "I may have a dispensary slot where a majority of the work will be processing prescriptions for Class B clinic and memory clinic." P2
4. "Sometimes we have to do on call as well." P3
5. "so quite often covering and supporting the dispensary staff and technicians." P7
6. "And so I have a full day dispensary cover that could either be inpatient or outpatient." P8
7. "I'll be involved in the dispensary and making sure the dispensary is running properly and potentially accuracy checking dispensed medication." P9
8. "I might then have a little look in the clinic and just see, you know, what's going on with the status of the nursing staff storing the medication." P10
9. "I might then go back to the pharmacy just to help out with what's going on with their supply." P10
10. "One could be like a dispensary day where you work in the dispensary to make sure that you know the work that's coming through." P11

Code: Managing supplies

1. "I then look at the nurse supply request that we get." P1
2. "I help with the logistics and supply of medication." P2
3. "I would first prioritise and any kind of supply of medication." P2
4. "Nurses will put nurse supply requests for medications... I'll ensure the wards have the medication." P5

5. “So providing medicines and advice for clinicians and patients and we run two pharmacies, we have a community.” P7
6. “Part of our job also being in dispensary purely just supply medication towards as required.” P8
7. "For the remainder of the week I spend some time in the pharmacy and clinically checking prescriptions and facilitating the supply functions." P10
8. "We've got a supply function of as well to make sure that medications reaching the wards and also. Making sure that the medicines management side of things are going well as well on the board." P11

Code: Discharge medication planning

1. "When I arrive at work, I checked to see if we got any discharges... if it’s a short-term leave, how many days? How many of this tablet do we need?" P1
2. "When we discharge a patient, we need to make sure that they often are safely discharged and they will need to be discharged into the community mental health team after an acute inpatient episode." P2
3. “We would be looking at the discharge medication.” P5
4. "I'll ask about things like discharges. If there's anyone that needs, you know, medication prepared in anticipation of discharge from the unit so that I can counsel them in advance." P10

Minor theme 1.4. Involvement in teaching, research, and quality assurance

Code: Audits and quality assurance

1. "There's also audits that we have to do." P3
2. "We do CD audits on a regular basis every two months." P4
3. “We do a lot of auditing that overall it will benefit the team.” P8

Code: Research and information support

1. "If they have any kind of queries that come from the medics, then... I'll go away and research what they need to know and come back to them." P1
2. "We’ll look at like evidence-based situations... NICE guidelines... recommendations from the RPS... Maybe we should try this." P1
3. "I show them the evidence for it... and encourage them to share their evidence if they have any... if they don’t have any evidence, maybe this isn’t a good idea." P1
4. "I'm also the clinical trials pharmacist at our hospital." P2
5. "As a pharmacist, we provide evidence-based recommendations with pros and cons to support decisions." P4

Code: Teaching, preceptorship and education

1. “I do training to the trainer year through HE about mental health medication...I do teaching to various undergraduate pharmacy and nursing courses about mental health medication." P9
2. "I work as a teacher practitioner, pharmacist and my role is split 50/50 between teaching at (mentions the university) and working in a mental health setting." P10

3. "I’m also the designated supervisor to review trainees." P2
4. "We have students from [name of university]...We have our own pre-reg trainees that are with us for the year…Sharing [with] them, building them, being a good role model for them, and then helping them, you know, be the best that they can be." P3
5. "We have clinical supervision once a month with our pharmacists where we have the opportunity to talk through difficult cases and experiences." P9
6. "Educating the trust emergency department about Clozapine and its serious side effects." P4
7. "A big part of my job is also educational." P8
8. “Educate our new nursing or new staff with regard to those changes that could be a new policy.” P8

Minor theme 1.5. Leadership and strategic roles

Code: Leadership roles

1. "I also do a bit of management of the rotor side of things." P3
2. "I'm so currently I'm a lead pharmacist, so I'm leading a team of other Band 7 pharmacists and technicians." P8
3. “A big fraction of my job would be organizing and coordinating my team.” P8
4. "And then line management of eight people." P9
5. "I'm also the lead for the area [ICU in mental health].” P11

Code: Policy development and implementation

1. "Recently this week just finished compliance aid guidance for use in the older adult service and also through our Community pharmacy." P7
2. "If we have a new medication or new guideline in place, then part of my job is to promote it on site." P8
3. “And then I'm involved in a variety of the governance, and operational roles...pharmacological Therapies Committee, approval of policies and new drugs...governance processes within the Secure Care service.” P9

Theme 2: Positive aspects and satisfaction

Minor theme 2.1. Satisfaction

Code: Satisfaction and recognition in job

1. "I get a lot more job satisfaction from working here... it's really good to be able to sit down... and figure out something that will actually benefit them." P1
2. "I think that in in terms of the role of a pharmacist, I think that's really, it's a good place for a pharmacist to be. And so I enjoy the work that I do." P1
3. "my main job satisfaction of [is] seeing the patients well again." P1
4. "I quite enjoy mental health work." P2
5. "It's most satisfying when they get well, but also we are able to put measures in place for them to stay well as well." P2
6. "The positives is the patient satisfaction and see them get better by the end of the thing." P3
7. "When you see a patient becomes well later on, you feel that satisfaction." P4
8. "Even just one of those in a career makes it all worthwhile." P7
9. "I think this area that I really love to walk in, it's very rewarding..." P8
10. "When we have pharmacists on the team, the patient intervention is there much quicker and more efficiently." P8

Code: Satisfaction with team and work environment

1. "If it becomes a bit of a worry, you can kind of bring someone else in just in case as well." P1
2. "Honestly, the people who work in mental health are probably some of the nicest people...they do involve as much of the team as possible at all times. P2
3. "It's actually very, very good rapport with both the MDT, the ward as well as the patients themselves." P2
4. "It creates kind of like a good bounce back and a bit of sense of community with your other mental health colleagues." P2
5. "If the team is cooperative, your day will go OK, I guess." P5
6. "I've got the backing of my team as well...we solve problems together in monthly meetings." P6
7. "The people I work with, I can see there is a shared value between me and my colleagues, which makes the job more rewarding." P8
8. "We're very supportive of each other…I've got lots of friends at work, not just work colleagues, and you know, I'm lucky to work with such a good team." P9
9. "My team and the culture of the teams that I work in—the pharmacy team alongside the medical teams and nursing teams, psychology, etc.—the approach and the culture, it seems to be a lot more empathetic." P10

Code: Peer learning

1. "It's quite nice to be able to build that rapport with the team... be able to bounce ideas off... have that relationship and it's really nice." P1
2. "There are so many specifics that you can learn from other people's practice in their own field." P2

3. "There was an issue that happened... So we learned from that, we discussed, and we learned from that." P3
4. "If I have to divert from guidance... I always seek senior advice." P5
5. “we have ongoing monthly supervision to make sure that we up to date with the with the latest training and requirement.” P8
6. “It may not help in the moment of the issue, but it helps us all to learn from each other." P9
7. "I think it's having really good peer support and that ability to be able to talk through difficult scenarios. Whether that's pharmacist peers or actually other medical peers." P9

Minor theme 2.2. Recognition of mental health and pharmacy services

Code: Recognition within team

1. "You feel valued with the role that I do." P3
2. "There is always an opportunity for me to promote myself and educate myself further." P8
3. "I feel proud of my work because I feel like with mental health, not a lot of people, as pharmacists, want to do it." P10

Code: Change in public attitude towards mental health

1. The attitudes in a younger generation are a bit changing. P2
2. It’s better than before... 5-10 years ago to now is better. Most people do understand that mental health is like physical health. P4
3. Since COVID, I think the whole British population are quite willing to talk about...the troubles that they’re going through. P6

Minor theme 2.3. Positive impact on patient

Code: Building relationships with patients

1. "If they become settled, you can then talk to them... after maybe a few days or a week, they might feel more settled and willing to talk." P1
2. “You can build a rapport with, you know, … the patients… figure out something that will actually benefit them…” P1
3. "It feels more personal and level that you can get to know the patient a lot more." P2
4. "Being able to calm them down and speak to them about their medication was really, really nice." P2
5. "Actually, I'm involved in patient care for a long period of time, and on a professional level, you build up that relationship with people." P9

Code: Witnessing patient progress

1. "It's really nice when you see someone when they've been so unwell... to get to a point where they can leave and they just seem a lot better." P1
2. "We do have patients that do come, they go, they go and they come back... it's quite nice when they do go and you see them responding well." P1

3. "Being able to know the patients and also being able to see the results of having done so much work for a patient at any point in time." P2
4. "Seeing that you helped someone…Put a smile on their face and give them their life back." P3
5. "It's really rewarding when you see your patients get better and get discharged." P5
6. "I'm always happy with it, and the patient was happy with it as well." P6
7. "It's a service whereby you can sometimes... you get something right, you're there in front of the patient and you can see the difference." P7
8. "It's also gives you option to educate and also learn more about the area that you've worked and you also see the patients progress." P8
9. "I'm very passionate about what I do and you also see patients' journey." P8
10. "It's really satisfying to see people make improvements, reach their recovery, and manage to move on to a really meaningful life after having been in a medium secure unit." P9
11. "When I know someone could go home and, you know, stay well and they've been given the tools to do that, then I feel that I've done something positive for them." P10
12. "So I feel proud because I'm able to make a difference to individuals." P10
13. "It's having those consultations that I find rewarding and helping that patient may come to decisions." P11
14. "Definitely the rewarding factor you get from seeing people get better." P11

Code: Promoting person-centred care and reassuring patients

1. "Making sure things are like patient-centred like person centred care... bring the patient in and actually get them involved with everything." P1
2. "There's a big push for like no decision about me without me... that's kind of put into play as well." P1
3. "I do feel like I get I'm able to give more input into patients." P2
4. "It's mostly focused on patient-centred care or person-centred care rather than what we think." P4
5. "Over the years, because I've been working in mental health for so long, just the opportunities to get the medication right for somebody, really individualize it, listen to them and equally help them to understand." P9
6. "You can have the best guidance in the world, but if the patient doesn't quite fit the criteria or doesn't meet the rules, you've got to actually look at the real person." P9
7. "We're putting the patient at the heart of everything... But obviously with mental health, it's not always clear cut. Look at that individual patient and try and fit into their needs." P11
8. "We see the patient look at sort of what plans we've made for medication, discuss that with the patients, they might have other stuff going on as well, but would obviously be intervened in the medication side of things." P11
9. "I'll help them give them the information so they can decide what choices they wanna make and provide them sort of information on side effects, things like that." P11
10. "You've had that consultation with the patient that you know there's a particular medication that they. You want them to try all the team wants them to try, but they've got reservations about it and you can go and clear those reservations up and give them reassurance that what they're thinking might not be the case." P11

11. “If you speak to them, you can reassure them about medications and stuff.” P11

Theme 3: Challenges and barriers

Minor theme 3.1. Stigma and attitudes towards mental health

Codes: Societal Stigma

1. “There is quite of negative view with mental health. Places because I think it's still quite taboo and so a lot of people obviously have kind of like this feeling towards mental health issues.” P1
2. "Especially when you're working with the elderly and Paediatrics...there’s a lot of stigma around it." P2
3. "There's stigma of just medication and then there's stigma of mental health medication." P2
4. "Her family refused to believe that it was an auditory hallucination and thought she was just a bad person." P2
5. "Generally mental health does have a stigma around it." P3
6. "The challenges were mostly the stigma” P4
7. "Stigma around mental health services is out there... maybe it has improved in the last few years, but it’s still there." P5
8. "There is a lot of stigma, lack of awareness, and just not being accepted by society." P8
9. “Unfortunately, there is a lot of stigma in our society around mental health." P8
10. "Some families and cultures don’t even recognise mental illness." P10
11. "I've done patient groups and stuff where they've recognised that there's stigma out there and they have spoken about the stigma they feel." P11
12. "I feel like stigma is getting less than what it used to be.” P11

Code: Fear of labelling and judgment

1. "They don’t want to be labelled with a diagnosis because of the negative connotations." P10
2. "People are scared, fearful, and there's still a lot of stigma involved with just mental health in general." P10
3. "A lot of them might be worried—'Oh, a mental health professional is seeing me, now I have to admit to whoever's next to me that I have a mental health problem or a mental illness.’" P10
4. “But they feel like there’s nothing wrong with them. They don’t want to be seen as anything different." P11

Code: Denial and reluctance to acknowledge mental illness

1. "A lot of people that are kind of in denial... probably because of the way that we’ve been brought up in the past." P1
2. "Some patients live in denial of their diagnosis because they don’t want to be diagnosed." P5
3. "People don’t want to admit and talk about...mental health problems, so we’re sort of hiding behind shadows." P6

Code: Negative perception

1. "I know the kind of things people would say about this building... call them nasty names." P1
2. "When you say I treat schizophrenic patients or patients with bipolar, then it becomes like, aren't you scared or isn’t that dangerous?" P2
3. "They (mental health patients) are seen as problems by other people." P7
4. "One of the challenges that we have is people's perceptions and experience of mental health services.” P9
5. “I know when I've had students before and they've been quite frightened to come to a locked ward.” P11

Minor theme 3.2. Role ambiguity and interdisciplinary collaboration

Code: Underrated role

1. "I think as a healthcare provider, pharmacists sometimes are underrated. We don't appreciate how much of an input we can have." P8
2. "As a pharmacist, when you go to a team, if you haven’t been introduced to that team properly or if they’re not very aware of what a pharmacy team can do, it can be quite challenging. They might not see insight into what a pharmacist does for the team." P8
3. "When we get people coming into the service—prescribers coming into the service—who haven't worked with such an established pharmacy team, sometimes they can be a little surprised at the level of input that pharmacy have." P9

Code: Interpersonal dynamics

1. “It’s better than it is in general... here you know who you’re gonna be talking to... but it can be a challenge if you’ve never covered a ward before." P1
2. "Sometimes you do come across... challenging colleagues... finding that balance between how you would like to communicate yourself to them." P1
3. “You might have kind of the idea of, oh, I think it should be this way. And they have a new idea...” P1
4. “Do you have any evidence for this…the responses that I've had in the past… well, the consultant said so… you can't really use that as an excuse if you're prescribing something… you need to have a rationale. If you were to go into a court of law, you can't just say, the consultant told me to do this.” P1
5. “Sometimes we may not all have the same ideas about a patient's care plan. One might feel he needs to go to a forensic or might feel he needs to, you know.” P3
6. "There may be disagreements within the team on recommendations... but as a pharmacist, you give evidence-based recommendations." P4
7. "You are not the person who has the final say... you work within the team." P5
8. "Sometimes it can be a lack of communication between different professions, that lack of awareness." P8
9. “it's just different profession might have different opinion, but how to justify it and how to professionally approaching it is important.” P8
10. “I pick my battles with this individual [prescriber] so that I don't constantly clash because we've got to work together.” P9

Minor theme 3.3. Educational and practice gaps

Code: Gaps in curriculum

1. "You're more focused on general health than kind of mental health... it is a big jump between coming out fresh and then going into mental health." P1
2. "We only have like a small part of mental health as part of the curriculum... the exam doesn't have that much with mental health." P1
3. "You will go out as a newly qualified pharmacist not knowing much about mental health medication…You only have maybe done a few lectures in one of your years." P5
4. "When I became a pharmacist in [year], I had no idea that you can get into mental health or that there is even a need for it." P8
5. "So many people come in thinking they don't need to know much about it, they can just learn on the job." P10
6. "I've had many stories from colleagues who are now teachers in my university who say their mental health experience was just being left to read some notes or do a case study...And the things that they were reading about, they just weren't mentally prepared to read about." P10
7. "When I came out after qualifying, I didn't know much about mental health at all until I actually went into it." P11

Code: Gaps in training

1. "lack of training resources." P2
2. "In practice, clinical supervision is good, but it's not very structured." P2
3. "But reality is they don't have the experience, and so they may not make good prescribers without that extra training." P7
4. "There is definitely a gap in our training services." P8
5. "I think it's just down to funding and training and skill mix staffing as well." P10

Code: Gaps in practice

1. "When mental health patients go into general hospitals, that side of it is completely ignored because pharmacists in different areas don't have that kind of breadth of knowledge across the two different domains." P7
2. "First of all, we need to make sure that we are up to date ourselves." P8
3. "We then have a large proportion of staff who don't feel confident in it, don't feel interested in it, and don't really want to get their hands dirty or involved." P10
4. "I think definitely there's gaps. I mean, it's improving. People are being exposed to more mental health and we're getting students in all the time." P11

Minor theme 3.4. Difficult patient interactions

Code: Difficulties in communication and engagement

1. "Depending on how unwell they are... you can't really talk to them... it's kind of finding that like how and where they are... is it even worth kind of troubling them and kind of distressing them by trying to ask these questions?" P1
2. "If nothing's kind of documented very well... you don't really have much to go on if the patient's too unwell to speak to." P1

3. "It’s difficult to communicate with patients with learning disabilities because they may not understand what you're saying." P4
4. "When they relapse and you’re unable to communicate with them, you feel you're giving treatment against their consent." P4
5. "Because obviously mental health patients, depending on their condition, vary very much at their level of engagement and the quality of the interaction. So it's not just straightforward explaining something to somebody." P7
6. "A lot of my men aren’t very well, so sometimes they’re just not quite there yet for the conversation." P10
7. “You can't always get that information [illness related] from the patient." P11
8. "With mental health… someone may look OK when actually there's a big risk element that you could quite easily miss." P11

Code: Managing expectations and conflicts with patients

1. "There’s expectations for you to be able to convince patients to take their medication." P2
2. "There is an expectation of such high rapport with the patients, you do kind of sometimes get conflicts with the patients." P2
3. "You get conflicts with the carers or family and friends of the patients as well about what is the right thing." P2
4. “Explaining that medications can be administered to them against their will is quite a difficult conversation to have." P2
5. “You get resistance from the patient…Sometimes you get families as well, so you're trying to get the patient to agree, but then the family gets involved." P4
6. "Some of it is related to the patient themselves because of a lack of insight into their mental health." P8

Code: Treatment refusals and non-adherence

1. "When they wanted to put the second antipsychotic, the patient refused to have it." P3
2. "We see relapse patients coming back because they stopped taking the medication due to certain side effects." P4
3. "Mental health is very much about the connections that are made and not being able to get to see the patients directly as much as we would like is probably one of the biggest regrets." P7
4. "Someone that is suffering from mental health, and that's one of the barriers that patients don’t take their medication." P8
5. "The vast majority of my patients are very opposed to being in hospital and don't particularly want to talk and engage about their treatment." P9
6. "They might not want to take the medication, things like that." P11

Code: Risks and emotional impact

1. "Sometimes getting harassed on the ward...you do sometimes get some racial or sexual remarks." P2
2. "It can impact your mental health because you are dealing with these things every day." P5
3. “We're dealing not just with somebody's severe mental illness, but the associated risk management and risk of reoffending." P9

4. "I work on a psychiatric intensive care unit, I am always... working alongside patients who have got a history of violence, aggression."
5. "That's sometimes, you know, mentally something that's quite scary." P10

Minor theme 3.5. Institutional challenges

Code: Workforce shortages and staffing

1. "we don't have the [human] resources, the people who are trained in mental health to be able to cover as much as we want you to give the amount of advice that we want to." P2
2. "We don't have as many trained mental health pharmacists." P2
3. "The national agenda for our mental health patients is sometimes we can't meet that because we don't have enough staff." P3
4. "Staff shortage is something that everywhere you come across... you won't be able to complete your work." P4
5. "We've really struggled to get people interpose. We are now literally 22 posts short of being full, which is a new record." P7
6. "We had a KPI setup which kind of fell by the wayside because we just didn't have enough staff to train the new staff in that level of detail." P7
7. "Most of the time there is a lot we can do. There are a lot of resources we can use, but you're only one person with all these challenges." P8
8. "We, like I say, lots of ideas and just not enough staffing." P9
9. "Staffing remains a concern, and we need to ensure a better skill mix for more effective mental health pharmacy services." P10
10. "So more staffing basically." P11

Code: Time management

1. "Systems in place are quite convoluted. It's quite difficult to know who to go to for what specific thing, for example." P2
2. "I think it's mainly, I would say, time management." P2
3. "That's why we need more time to do our job." P3
4. "Finding the time to be able to deliver all these tasks and all these plans that you have in that given time can be challenging." P8
5. "If you've got more like resources plugged into mental health, then you've got more time to sit down and the patient gets to know you a bit more and stuff." P11

Code: Work complexity and load

1. "The challenges would be like I said is a lot of in-depth work, so lots of research…Sometimes it is a lot of just your own research that you have to do to see what's best for the patient." P2
2. "There's a lot of nuances that you will only learn through practice." P2
3. "I…feel like we are trying to very much meet those standards and it's quite difficult, the workload. So while the intention is there, the jump is not high enough." P2
4. "We don't have as many guidelines as other areas of work, so a lot of it does have to come from compilations of research." P2
5. "I feel like I don't get to do everything I want to do for a patient... there's just too many patients that you can't physically get round everyone." P11

Code: Challenges in treatment and prescribing

1. "Sometimes you do encounter a lot of unlicensed usage." P2
2. "Reluctance of prescribers outside of mental health to prescribe or de-prescribe mental health medications." P2
3. "When I was in (name of workplace), we got quite a lot of calls saying this GP won't do this for me, my child doesn't have an antipsychotic anymore, please help kind of thing." P2
4. "A lot of the medications can be reduced after they're better, and they're not reviewed." P3
5. "They (prescriber pharmacists) will not be ready to prescribe in mental health when they graduate...it takes years of practice and learning." P5
6. "We mustn't underestimate how powerful the drugs are that we're giving people, and we've got to try and keep that balance right." P9

Code: Funding

1. "There are some gaps within funding, for example, and we want to get a drug in." P3
2. "Ongoing challenge is going to be funding because obviously things like the 20 pharmacists... but the funding's being cut..." P7
3. "Gotta be honest, funding is one of them." P7
4. "My son being a physio student gets the £5000 a year learning support and gets travel expenses for placements. Physio, OT, and nursing students get that. Pharmacy doesn't." P9
5. "If we've got two students for a morning coming to an MDT with us, that's £28.00 that we get paid. That's not enough for us to be able to try and support and develop." P9
6. "It's very underfunded relative to the numbers of people." P10

Theme 4: Views and recommendations

Minor theme 4.1. Views about supplementary courses

Code: Shortcomings in current prescribing courses

1. "In theory, pharmacists working in whichever domain can move between the others more effectively." P7
2. "What we should do is either a general certificate and then the mental health diploma, or the Mental Health Certificate and then a general diploma to finish off—it'll give them a much better breadth of knowledge." P7
3. "I found that the prescribing course was heavily weighted to physical things, which are important, but there just wasn't enough in there for me to then go back to my practice and say, 'OK, well, I learned this.'" P10
4. "I didn't feel that the course itself really equipped me." P10
5. "Whereas if it had a mental health extension or component that was more in-depth, it would allow me to feel more confident as a prescriber at the get-go." P10

Code: Importance of supplementary training and certification

1. “I think there are kind of areas which can be kind of optimised with training… I have undertaken the (name of certification) and (name of certification).” P1
2. "Being prescribers right after you finish pre-reg, it’s absolutely wild…specialising pharmacists so their IPs are actually useful is probably the best bet." P2
3. “When you do that [postgraduate certification], then you kind of get to learn about a lot of different things within mental health. And then you can apply that to the get the resources, you get the knowledge.” P3
4. "For mental health pharmacists, I think they should definitely, just as any other field, they should definitely do the IP course." P3
5. "Specialised training ensures you are competent to provide and optimise the service." P5
6. "I have done the certificate in (name of certification), which basically just means a lot of work around the various typical mental health conditions—dementias, mood disorders, psychosis."
7. "We now automatically put our junior pharmacists through that once they've done their pre-reg and qualified." P7
8. “Then I did my certificate in mental health, followed up by a diploma in a psychiatric therapeutic, and I did my prescribing course in the same setting.” P8
9. "There are certificates and training that people can do to make themselves more trained in this field." P8
10. "I think it's about being adequately equipped with teaching, not just at an undergraduate level, but also encouraging more people to take it seriously and do the postgraduate certificate, at least in mental health therapeutics." P10
11. "Then, when people come out as newly qualified, it's about getting pharmacists onto specialist courses." P10
12. "There should be bespoke training, maybe on core conditions that they’re [pharmacy technicians] likely to encounter in mental health settings." P10
13. "I think ensuring that people who are working in mental health as pharmacists go further in their training is important." P10

Code: Barriers to using independent prescribing qualification in practice

1. "We don’t have capacity at the moment for pharmacists having clinics and having their own caseload." P9
2. "I find that a lot of people don't actually use the [prescribing] qualification." P10
3. "The other pharmacists haven’t been using it yet because of lack of confidence or lack of space for them to be able to use it in their day-to-day work." P10
4. "At most, you just end up changing the old prescription on behalf of a doctor, which is no different to what we were doing before the qualification." P10

Minor theme 4.2. Views about prescribing for pharmacists

Code: Potential benefits of having prescriber-ready pharmacists

1. "If they were independent prescribers... it would reduce the workload of [clinicians] and maybe the mental health service would be better equipped." P1
2. "That's [prescribing] going to be even more important for the prescribing pharmacists in 2026." P7

3. "I think there’s a lot that pharmacists can offer when we wear our prescriber hats because we have a very different perspective." P10

Code: Concerns about readiness for prescribing after MPharm

1. "Some will not want to prescribe because they're not confident; some are going to be overconfident and wanting to prescribe without any kind of experience in a particular area." P7
2. "Pharmacists coming out of university won’t have had access to mental health services, but the expectation will be they’ll come in ready to prescribe without that understanding." P7
3. "We’re already looking at how we can delay prescribing until they [newly qualified pharmacists] ‘ve done at least the certificate." P7
4. "Some of them will be very confident. Some of them will be not at all confident, and we've gotta find that nice path down the middle whereby they are sufficiently competent and confident." P7
5. "But it seems to be they're coming out as generalists, and if we compare a pharmacist to a generalist junior doctor or a trainee GP, they've had an additional two or three years of training before they were allowed to do that. And it does worry us quite a lot." P7
6. "People who don't do the IP course now—they're going to be up against in two years...Once that happens and the younger people come out qualified, there's gonna be a push to try and squeeze down pay scales to make it more cost-effective." P7

Code: Lack of experience and confidence among newly qualified pharmacists

1. “I have concerns for people that have just qualified from pharmacy school to become a mental health prescriber. I don’t think it’s got enough insight or enough in-depth information to make you a good prescriber when you haven’t had enough experience." P8
2. "I'm not too keen on people going directly from university to prescribing. But after they have enough experience, most definitely yes." P8
3. "I found it still really scary to be an independent prescriber. It's a big shift in responsibility. I'll admit I have concerns about a newly qualified pharmacist prescribing to the sorts of patients that I look after." P9
4. "I think that would be not having enough experience in mental health [at start]… how are you confident [in prescribing]?" P11
5. "But that small [confidence] come with experience rather than my prescribing ability." P11

Minor theme 4.3. Recommendations

Code: Increasing awareness in the community

1. “Having more of awareness of mental health and kind of viewing it as you know, much the same as physical health, as some mental health, you know, things like that.” P1
2. "If we have a bigger drive with treating people out in the community... it would avoid admissions." P1
3. "A bit more advertising of the opportunities that are available in mental health." P2

4. "We have to do a lot of work as healthcare providers to raise awareness that mental health illness is like any other illness that needs to be treated." P8
5. "I don't think there's enough promotion of mental health to new pharmacists. We should raise awareness a bit more about the area that people can work in mental health." P8
6. "I would definitely recommend making more events around mental health services. Mental health is very hidden at the moment, so we should have people from mental health coming to university to have a talk. There should be a stand at job fairs promoting mental health pharmacy." P8
7. "Not many people who are passionate about mental health and really want to come and work in mental health." P9
8. "It's about making sure whether it's about specialist mental health services or just in any pharmacy setting, you are going to be coming across people with mental health issues." P9
9. "So it's making sure that people don't think that you can only know about mental health if you work in a mental health trust." P9
10. "It's very much recognising the value of mental health pharmacy staff." P9
11. "We know that it's important to increase access to mental health services in different areas." P10
12. "Most people don't have much exposure to mental health hospitals." P10
13. I would say that it's all good and well talking about getting the students to know more about mental health, but they need to get their own knowledge up to scratch. P10

Code: Increasing content in university curriculum

1. "Having more lectures, having more of an awareness, having more kind of promotion about being a mental [health] pharmacist." P1
2. "I would want the university to encourage them to look into it [mental health]." P4
3. "There should be more lectures distributed, not just in one year... it's very, very heavy content." P5
4. "There should be a module in university by third or final year to give a bit more insight into mental health." P8
5. "We should have a lecture where a mental health pharmacist comes to the university and talks about mental health—what it covers, what job opportunities there are, the challenges, and the positives and negatives." P8
6. "A lot of our challenges are about staffing, but you can't get more staff unless you support the undergraduates through and get them interested in mental health pharmacy." P9
7. "In order to improve things, it comes down to what pharmacists get taught at a degree level." P10
8. "Even if they aren't interested in mental health, to then talk to students and encourage them to go into it as a field, they should be interested in mental health because there's a duty of care to students." P10

Code: Exposure to mental health in training and placements

1. "If it's incorporated into the course [MPharm] a bit more... it would entice more people to then go out and become mental health pharmacists. And it wouldn't be viewed as like the taboo." P1

2. "I would recommend that undergrads do placements in mental health hospitals…Placement can really make you understand what real life is... maybe two weeks in the second year and two weeks in the third year." P5
3. "People have a poor understanding of it, but I think things like the student placements help...All of our pharmacy students at [name of the institute] and the other universities here have to do a mental health placement." P7
4. "You do cover basics in university, but they are not really mental health-focused as such."
5. "We cover anxiety, depression, insomnia, and epilepsy in pharmacy school, but it's not in-depth." P8
6. "I think all pharmacists should have a placement in mental health. There are some pre-regs that have mental health placements as one side of the pre-reg year, which I think is an excellent way of introducing mental health to new pharmacists." P8
7. "The experience we're seeing about undergraduate, and placements is improving both in terms of knowledge about medication and also just confidence in approach to people." P9
8. "I'm really, umm, passionate about supporting students because I did a two-week placement in mental health in my training year… and just really fell in love with the place." P9
9. "Simulated mental health environments are something that I've worked on more, just giving those opportunities to discuss things you might need to know when talking to a patient...Providing a safe space for the students to ask questions as well and to develop healthier attitudes." P10
10. "If they're thinking about going into mental health, it's good to be able to get access via placements to get a feel of what it's like." P10
11. "I wish that we were able to do better with the training early on so that we could then have people in post when they're graduating from university." P10
12. "Just having more exposure to it, so having patients and students going to see patients a lot more and getting that feel for it." P11

Code: Having structured support in mental health

1. "Having specific entryways to get into mental health would be very good." P2
2. "It would be good for a more systematic kind of mentorship…Having a more experienced Band 8 pharmacist lead and continuously monitor someone who has just entered a mental health field." P2
3. "A model where you have a senior pharmacist and a junior pharmacist covering every single acute mental health ward would be the best." P3
4. "I think that the support should be in place—if they're interested in a mental health placement, they should discuss that with someone early on." P10

Code: Increase in NHS staffing

1. "More staffing would be nice…more staff...a higher ratio of healthcare professionals per patient." P2
2. "If you could change anything about the mental health care... have more pharmacists." P3
3. "Supporting newly qualified pharmacists as prescribers and essentially supervising pretty much everything that they do, I think that staffing challenge is gonna be significant." P9
